# Development of multi-epitope vaccines against the monkeypox virus based on envelope proteins using immunoinformatics approaches

**DOI:** 10.3389/fimmu.2023.1112816

**Published:** 2023-03-13

**Authors:** Caixia Tan, Fei Zhu, Pinhua Pan, Anhua Wu, Chunhui Li

**Affiliations:** ^1^ Department of Infection Control Center of Xiangya Hospital, Central South University, Changsha, Hunan, China; ^2^ National Clinical Research Center for Geriatric Disorder, Xiangya Hospital, Changsha, Hunan, China; ^3^ Department of Respiratory Medicine, National Key Clinical Specialty, Branch of National Clinical Research Center for Respiratory Disease, Xiangya Hospital, Central South University, Changsha, Hunan, China; ^4^ Center of Respiratory Medicine, Xiangya Hospital, Central South University, Changsha, Hunan, China; ^5^ Hunan Engineering Research Center for Intelligent Diagnosis and Treatment of Respiratory Disease, Changsha, Hunan, China; ^6^ Clinical Research Center for Respiratory Diseases in Hunan Province, Changsha, Hunan, China

**Keywords:** monkeypox virus (MPXV), multi-epitope vaccine, molecular docking, molecular dynamics (MD) simulation, immunoinformatics

## Abstract

**Background:**

Since May 2022, cases of monkeypox, a zoonotic disease caused by the monkeypox virus (MPXV), have been increasingly reported worldwide. There are, however, no proven therapies or vaccines available for monkeypox. In this study, several multi-epitope vaccines were designed against the MPXV using immunoinformatics approaches.

**Methods:**

Three target proteins, A35R and B6R, enveloped virion (EV) form-derived antigens, and H3L, expressed on the mature virion (MV) form, were selected for epitope identification. The shortlisted epitopes were fused with appropriate adjuvants and linkers to vaccine candidates. The biophysical andbiochemical features of vaccine candidates were evaluated. The Molecular docking and molecular dynamics(MD) simulation were run to understand the binding mode and binding stability between the vaccines and Toll-like receptors (TLRs) and major histocompatibility complexes (MHCs). The immunogenicity of the designed vaccines was evaluated via immune simulation.

**Results:**

Five vaccine constructs (MPXV-1-5) were formed. After the evaluation of various immunological and physicochemical parameters, MPXV-2 and MPXV-5 were selected for further analysis. The results of molecular docking showed that the MPXV-2 and MPXV-5 had a stronger affinity to TLRs (TLR2 and TLR4) and MHC (HLA-A*02:01 and HLA-DRB1*02:01) molecules, and the analyses of molecular dynamics (MD) simulation have further confirmed the strong binding stability of MPXV-2 and MPXV-5 with TLRs and MHC molecules. The results of the immune simulation indicated that both MPXV-2 and MPXV-5 could effectively induce robust protective immune responses in the human body.

**Conclusion:**

The MPXV-2 and MPXV-5 have good efficacy against the MPXV in theory, but further studies are required to validate their safety and efficacy.

## 1 Introduction

Monkeypox is a zoonotic disease caused by an enveloped double-stranded DNA (dsDNA) virus known as monkeypox virus (MPXV), which belongs to the Orthopoxvirus genus of the Poxviridae family (1). Most cases of monkeypox present with mild disease symptoms, such as fever, rash, lymphadenopathy and intense asthenia, but certain population groups (children, pregnant women, and immunocompromised individuals) may suffer from severe disease or complications, including secondary bacterial infections, pneumonitis, respiratory distress, sepsis, encephalitis and even loss of vision following cornea infection (2). The MPXV was first identified in humans in 1970 (3). Since then, sporadic cases have been detected from time to time, but most of them were confined to West and Central African nations, or have a history of travel to African countries or exposure to imported animals (3). However, since May 2022, there has been a sharp increase in the number of MPXV infections worldwide, with the majority of confirmed cases occurring in countries that have not historically reported human monkeypox, and there are no obvious links between the documented cases in different countries (4). As of 13 November 2022, a total of 79,411 laboratory-confirmed monkeypox cases and 50 deaths have been reported in 109 countries, according to WHO (5). However, aside from several antiviral drugs for smallpox (Tecovirimat, cidofovir, and brincidofovir) that can be used to treat monkeypox under certain conditions, there is no proven post-exposure treatment (6). Hence, an effective vaccination against MPXV is warranted.

To date, there is no specialized vaccine against MPXV, but a small study based on previous African data estimated that the smallpox vaccine may provide approximately 85% cross-protection against MPXV (7). There are several available smallpox vaccines, including ACAM20, MVA-BN and LC16. ACAM2000, a live vaccinia virus-based preparation, and MVA-BN, a non-replicating smallpox vaccine, were both licensed by the US Food and Drug Administration (FDA) for active immunization against smallpox (8). However, the former may lead to secondary vaccinia infection in vaccine recipients and those in close contact with them, and some vaccine recipients may develop myocarditis or pericarditis (8). Although data from human immunogenicity trials and pre-clinical studies suggested that the later might offer some protection against MPXV in humans, no large-scale clinical study has been conducted to determine its actual level of protection against monkeypox infection in humans (8). Similar to MVA-BN, LC16 is a live, non-replicating attenuated vaccine, that was approved for the prevention of monkeypox in August 2022 in Japan (8). Also, its efficacy against monkeypox is extrapolated from data from animal studies rather than direct human trials. So, in short, there are research gaps in the monkeypox vaccine research that needs to be filled.

The multi-epitope vaccine, a novel type of vaccine developed in recent decades, has shown good immune effects against microbial infections and has fewer side effects than traditional vaccines in animals and early clinical trials (9). The application of immunoinformatics in vaccine development offers a more effective, cost-effective, and time-saving approach to developing vaccines for infectious diseases (10). The selection of targets is crucial for developing effective multi-epitope vaccines. Since no specific receptor for MPXV on the host cell membrane has been found so far, several envelope proteins, playing a key role in the invasion of host cells by MPXV, may be attractive targets for MPXV vaccine development (11, 12). A35R, homologous to vaccinia virus A33R, is an envelope glycoprotein of EV and is required for the formation of actin-containing microvilli and virion transmission between cells (13). B6R, located both on the membranes of infected cells and on the enveloped virion (EV) envelope, is similar to the complement control protein-like B5R of the vaccinia virus. Mice immunized with a subunit vaccine containing B5 could produce anti-B5 antibodies capable of neutralizing extracellular viruses, and destroying infected cells, according to Paran et al (14). Of note, existing neutralization tests demonstrate only the antibody reaction to B5 is available for neutralizing EV (14). Studies have shown that mice immunized with H3L can produce anti-H3L antibodies that protect them from a lethal dose of vaccinia virus (15). In addition, H3L can be recognized as an antigen by CD8+ T cells (16). Given the above, A35R, B6R, and H3L are attractive targets for MPXV vaccine development.

A recent study found that MPXV has an high mutation rate, which was 6 to 12 times higher than previously believed, and some of these mutations led to stronger MPXV infectivity (17).To provide broader protection against different strains of MPXV, three reference strains from three different epidemic phases of monkeypox were selected as the research objects, and the A35R, B6R and H3L of virus strains were selected as the protein targets to develop a promising multi-epitope vaccine using the immunoinformatics approaches.

## 2 Methodology

### 2.1 Data retrieval and sequence alignment

The amino acid sequences of A35R, B6R and H3L of three strains (Zaire-96-I-16, 2001, GenBank: GCA_000857045.1; MPXV-UK_P2, 2018, GenBank: MT903344.1; 2022 reference strain, GenBank: GCA_014621545.1) were obtained in FASTA format from National Center for Biotechnology Information (NCBI) (https://www.ncbi.nlm.nih.gov/) database. To identify the common and specific sites of the single protein from different strains, we performed alignment of the target protein sequences from three objective MPXV strains using the Clustal Omega program (https://www.ebi.ac.uk/Tools/msa/clustalo/) available on the EMBL-EBI server. The visualization of the results of sequence alignment performed by Jalview v 2.11 software (18).

### 2.2 Prediction and selection of epitope

#### 2.2.1 Prediction and selection of cytotoxic T lymphocyte epitope

The NetCTL 1.2 server (https://services.healthtech.dtu.dk/service.php?NetCTL-1.2) (19) was utilized to predict the Cytotoxic T Lymphocyte (CTL) epitopes restricted to 12 major histocompatibility complex (MHC) class I supertypes (A1, A2, A3, A24, A26, B7, B8, B27, B39, B44, B58, and B62). This method combines the prediction of peptide MHC class I binding, proteasomal C terminal cleavage and Transporter associated with Antigen processing (TAP) transport efficiency (19), and only epitopes with a combined score ≥0.75 were chosen for antigenicity testing using the VaxiJen v2.0 server (http://www.ddg-pharmfac.net/vaxijen/VaxiJen/VaxiJen.html) (20). VaxiJen allows antigen classification entirely based on protein physicochemical properties, overcoming the limitations of alignment-dependent methods. The accuracy of the VaxiJen in predicting the protective antigen of the virus is 70% (20). To minimize the toxicity and allergenicity of the vaccine and improve the safety of the vaccine, ToxinPred2 (https://webs.iiitd.edu.in/raghava/toxinpred2/algo.html), with an accuracy of 95.54% (21), and AllerTOP v. 2.0 (https://www.ddg-pharmfac.net/AllerTOP/index.html) (22), with an accuracy of 85.3%, were used to predict the toxicity and allergenicity of the epitopes, respectively.

The strength of immunogenicity reflects the ability of the antigen to induce an immune response. Class-I Immunogenicity tool hosted in IEDB (http://tools.iedb.org/immunogenicity/) (23) is considered to be able to predict the immunogenicity of peptides to some extent. So, the antigenic epitopes were further submitted to the Class-I Immunogenicity tool to check their immunogenicity. T cells require the detection of epitopes on MHC molecules to be activated and to exert their effector activity. The Tepitool tool (http://tools.iedb.org/tepitool/) (24) provided on Immune Epitope Database (IEDB) helps to predict the affinity between the peptide and class I human leukocyte antigen (HLA) alleles, and the IC50 is an indicator to evaluate the binding affinity of the HLA molecule to a peptide. Hence, The CTL epitopes with antigenicity, immunogenicity, non-allergenicity and non-toxicity were submitted to the Tepitool tool, and the default threshold IC50 (affinity) ≤ 500nM was used as the screening criteria.

#### 2.2.2 Prediction and selection of helper T lymphocyte epitope

The NetMHCIIpan-4.1 server (https://services.healthtech.dtu.dk/service.php?NetMHCpan-4.1) (25), appearing stronger predictive power for the prediction of HTL epitopes and class II MHC molecules compared to other servers, was employed to predict the (Helper T Lymphocyte) HTL epitopes that possess a good affinity to 14 HLA class II alleles (HLA-DRB1*01:01, HLA-DRB1*03:01, HLA-DRB1*04:01, HLA-DRB1*04:04, HLA-DRB1*04:05, HLA-DRB1*07:01, HLA-DRB3*02:02, HLA-DRB1*09:01, HLA-DRB1*11:01, HLA-DRB1*13:02, HLA-DRB1*15:01, HLA-DRB3*01:01, HLA-DRB4*01:01, HLA-DRB5*01:01) covering 95% of the world’s the population. For each HTL epitope, the IC50 and percentile rank were determined, and the epitopes with an IC50 ≤500 and a percentile rank ≤ 5% were selected for antigenicity, toxicity and allergenicity prediction *via* VaxiJen v2.0, ToxinPred2 and AllerTOP v. 2.0 servers, respectively. Interferon-gamma (IFN-γ) plays an essential role in viral clearance and the activation of the host immune response (26). In addition to regulating B cell growth and immunoglobulin secretion, interleukin 4(IL-4) also influences T cell differentiation (27). Interleukin 2(IL-2) is also a multifunctional cytokine that helps T cells grow, proliferate, and differentiate and potently enhances the function of B-cells (28). Hence, the selected HTL epitopes were further submitted to IFNepitope (http://crdd.osdd.net/raghava/ifnepitope/) (29), IL4Pred (http://crdd.osdd.net/raghava/il4pred/) (30) and IL2Pred (https://webs.iiitd.edu.in/raghava/il2pred/) (31) servers to assess their potential to induce IFN-γ, IL-4, and IL-2 secretion, and only epitopes that induce IFN-γ production and at least IL-2 or IL-4 production would be ultimately considered for vaccine construction.

#### 2.2.3 Prediction and selection of B-cell epitope

The ABCpred (https://webs.iiitd.edu.in/raghava/abcpred/) was applied to predict linear B-cell epitopes based on recurrent neural network (RNN). The 16 amino acids were selected as the window length (32). As with T-cell epitopes, the antigenicity was checked by VaxiJen v2.0, and only epitopes with an antigenicity over 0.8 were further submitted to AllerTOP v. 2.0 and ToxinPred2 servers to check their allergenicity and toxicity, respectively.

#### 2.2.4 Population coverage prediction of T cell epitopes

Population coverage in different regions is one of the important indicators in measuring the effectiveness of the vaccine. And its level size is mainly affected by the distribution and expression frequency of the HLA alleles that bind to the multi-epitope vaccine. To increase the population coverage of limited length multi-epitope vaccine, we calculated the world population coverage for each selected CTL and HTL epitope using the IEDB’s population coverage tool (http://tools.iedb.org/population/) (33), and only epitopes with a world population coverage greater than 5% were subjected to further analysis.

#### 2.2.5 Homology analysis of selected epitopes with human proteomes

The Peptide match server can quickly and accurately match the submitted peptides with the UniProtKB Human complete proteome (34). To avoid inducing host autoimmune diseases and cross-reactions, each selected epitope was further submitted to the Peptide match server (https://research.bioinformatics.udel.edu/peptidematch/index.jsp) (34) to check their homology to the human proteome, and only the epitopes are non-homologous to the human proteome were selected for vaccine construction.

### 2.3 Molecular docking of T-cell epitopes with major histocompatibility complex molecules

MHC molecules are essential for presenting the pathogen-derived peptides to the respective T cells. Thus, it is necessary to perform molecular docking between T-cell epitopes and MHC molecules to understand the possible binding mode between them. First, the PEP-FOLD 3.5 server (https://bioserv.rpbs.univ-paris-diderot.fr/services/PEP-FOLD3/) (35)was used to model the three-dimensional (3D) structure of T-cell epitopes as the ligand, and RCSB Protein Data Bank (RCSB PDB) website (https://www2.rcsb.org/search/advanced/sequence) (36) was employed to acquire the 3D structure of MHC molecules as for the receptor. For some class I and class II MHC molecules for which the 3D structures are unavailable on the RCSB PDB website, we use the 3D structures of HLA-A*02:01 and HLA-DRB1*01:01 as the substitutes, respectively. Then, the energy minimization of receptors and ligands was carried out by Swiss-PdbViewer PDB viewer software (37), and following that, the molecular docking was run by Autodock Vina software hosted in PyRx v0.8 (38). AutoDock Vina, a classic molecular docking tool, has better performance for the prediction of docking between small molecules and proteins (39). The docking results were further analyzed using LigPlot+ v.2.2 software (40). Furthermore, to comparatively evaluate of binding affinity between the selected epitopes and MHC molecules, the molecular dockings between seven experimental verified antigen epitopes of poxvirus and HLA-A*02:01 molecules were selected as the positive controls.

### 2.4 Design of multi-epitope vaccine constructs

All T-cell and B-cell epitopes that satisfied the filtration criteria were incorporated into the construction of the multi-epitope vaccine. The linker is an essential part of recombinant fusion proteins, which is capable of forming effective epitope conjugation between epitopes and allowing the independent immunological activities of the epitopes (41). Low immunogenicity is a major deficiency of multi-epitope vaccines. According to the previous study, a suitable adjuvant molecule can significantly boost the peptide vaccine’s immunogenicity and longevity (42). 50s ribosomal protein L7/L12 from Mycobacterium tuberculosis is capable of promoting DC maturation and diverse pro-inflammatory cytokine production *via* interacting with TLR4, which helps enhance cellular immunity (43). In addition to direct antiviral activity, β-defensins can regulate adaptive immunity to viral infection by recruiting naive T cells and immature dendritic cells (DCs) to the infected site (44). LT-IIC, a type II heat-intolerant enterotoxin (HLT) produced by Escherichia coli (*E.coli*), is an attractive adjuvant with the unique property of enhancing the body’s humoral and cellular immune responses to co-administered antigens (45). The Cholera Toxin B subunit (CTB) is also considered an attractive adjuvant due to its ability to efficiently bind to antigen-presenting cells and enhance the stability and half-life of the entire vaccine molecule (46). RS09, an LPS peptide mimic, is an agonist of TLR4. When used as an adjuvant in certain vaccines, it is capable of resulting in stronger immune activation and higher antibody production (47). All five adjuvants have been widely used in the design of multi-epitope vaccines in silico (48–51) and have been shown to stimulate the host’s immune response in animal experiments (43, 45, 52–54). So, in this study, each of the five adjuvants was added to the N-terminus of the vaccine using EAAAK liners, resulting in the formation of five vaccine constructs. Afterward, the PADRE sequence was linked to the adjuvant with the help of the EAAAK linker. When added to the vaccine construct, the PADRE, which can bind to most common HLA-DR molecules with high affinity, has been shown to optimize antibody responses and deliver significant helper T-cell activity *in vivo (* 55). The CTL epitopes were connected by AAY linkers, the HTL epitopes were linked using GPGPG linkers, and the B-cell epitopes were spaced with the help of KK linkers. The last CTL epitope was linked to the first HTL epitope and the first HTL epitope was connected to the first B-cell-epitopes using the HEYGAEALERAG linker. Finally, the “6xHis tag” was appended to the C-terminal of the vaccine construct *via* the RVRR linker to facilitate protein purification but not affect the functionality of the fusion protein.

### 2.5 Prediction of biophysical and biochemical features of the multi-epitope vaccine constructs

#### 2.5.1 Prediction of antigenicity, allergenicity, toxicity and physicochemical properties of the vaccine

To determine the ability of the vaccine constructs to bind to immune cell receptors (antigenicity), both the VaxiJen and the ANTIGENpro (http://scratch.proteomics.ics.uci.edu/) servers were employed. The allergenicity of the vaccine was assessed using AllerTOP v. 2.0 server. The vaccine’s toxicity was predicted by the ToxinPred2 server. The physicochemical properties of vaccine constructs, including molecular weight, the number of amino acids, theoretical isoelectric point (pI), estimated half-life, instability index, aliphatic index, and grand average of hydropathicity (GRAVY) index, were predicted using ProtParam tool (https://web.expasy.org/protparam/) (56).

#### 2.5.2 The prediction of transmembrane domains, probability of solubility, and signal peptide sequence of the vaccine

Since most amino acids constituting transmembrane region proteins are hydrophobic amino acids and membrane proteins cannot be expressed in prokaryotic expression systems, it is necessary to predict the transmembrane sequence of vaccines. The DeepTMHMM (https://dtu.biolib.com/DeepTMHMM) server, which is the most complete and best-performing method for the prediction of the topology of both alpha-helical and beta-barrel transmembrane proteins (57), was employed to predict the transmembrane domains of the vaccine. Many experimental studies are hampered by protein insolubility. The SOLpro server (http://scratch.proteomics.ics.uci.edu/) (58) can accurately predict the probability of solubility in *E. coli* of the protein upon overexpression. So, all designed vaccines were submitted to the SOLpro server to predict their solubility. To avoid the amino acid sequence of the vaccine from being cut off by signal peptidase as a signal peptide, the signalP-6.0 online server(https://services.healthtech.dtu.dk/service.php?SignalP-6.0) (59) was utilized to predict whether a vaccine has signal peptides, as well as the location of their cleavage sites.

### 2.6 Secondary structure prediction, tertiary structure modeling, refinement, and validation

Out of all vaccine constructs, only antigenic, non-allergenic, non-toxic, stable, hydrophilic, thermostable, without transmembrane domains and signal peptide sequence, and highly soluble constructs were further submitted to the PSIPRED 4.0 server (http://bioinf.cs.ucl.ac.uk/psipred/) to obtain its secondary structure. The PSIPRED 4.0 server performed protein secondary structure prediction based on position-specific scoring matrices with a prediction accuracy of over 84% (60). The tertiary structures of the vaccine constructs were modeled using the Robetta server (http://robetta.bakerlab.org). Based on the fact that a confident template for the putative domains of a submitted sequence is available or unavailable, this server performs comparative modeling or *de novo* modeling, respectively (61). And this server will generate five models of each submitted sequence. Then, the optimal crude 3D models of each vaccine sequence were submitted to the GalaxyRefine module placed in the GalaxyWEB server(https://galaxy.seoklab.org/), which can rebuild unreliable loops or termini of the initial model structures using an optimization-based refinement method and further generate five refined models of each crude model (62). Referring to the criterion that the better model with a lower MolProbity score, we picked the best 3D refined model for each vaccine construct and submitted it to the ERRAT tool, PROCHECK tool of structure validation service SAVES v6.0 (https://saves.mbi.ucla.edu/) and ProSA-Web server (https://prosa.services.came.sbg.ac.at/prosa.php) for quality verification. The overall quality factor generated by the ERRAT program is the number of non-bonded interactions (side chains) formed between pairs of different atomic types within 0.35nm, and models with an overall quality factor value greater than 85 are generally considered to have good quality (63). The Ramachandran plot generated by the PROCHECK tool checks the stereochemical quality of the protein structure, and a better model has more residues in the Ramachandran-favored region and fewer residues in the Ramachandran-disallowed region. The Z-score predicted by the ProSA-Web server reflects the overall quality of the model, and a positive Z-score indicates the input structure has problematic or erroneous parts (64).

### 2.7 Molecular docking

Stable interaction between the vaccine candidate and immune receptor is an important precondition to generating successful immune reactions. The TLRs not only hold a vital position in the first line of defense against pathogens but also play a vital role in linking innate immunity with adaptive immunity (65). Previous studies revealed that TLR2 and TLR4 can induce antiviral immune responses by detecting the virus coat proteins (66, 67). Class I and class II MHC molecules help the CD4+ T and CD8+ T cells recognize foreign antigens(vaccine). Therefore, the molecular docking of the vaccine candidates with TLRs (TLR2, TLR4) and MHCs (HLA-A*02:01, HLA-DRB1*01:01) was performed using the ClusPro 2.0 server (https://cluspro.bu.edu/login.php) (68). Since 2004, ClusPro has consistently been one of the best docking servers as demonstrated in Critical Assessment of Predicted Interactions (CAPRI), providing high predictive performance for the docking of protein-protein complexes (68). In each docking case, a total of thirty models were generated, and ten models with highly populated clusters were presented. Then, the best model in each docking case was further submitted to the HADDOCK 2.4 server (https://wenmr.science.uu.nl/haddock2.4/) (69), which is capable of refining the complex structures by rearranging the side-chain in the restricted interface and optimizing soft rigid-body, for refinement. After refinement, the docked models were submitted to the PRODIGY server (https://bianca.science.uu.nl/prodigy/) (70) for predicting binding energy. Furthermore, the PDBsum server (http://www.ebi.ac.uk/thornton-srv/databases/cgi-bin/pdbsum/GetPage.pl?pdbcode=index.html) (71) was utilized to investigate interacting residues between docked chains (i.e., the vaccines, the TLRs and MHCs). The PyMOL v2.5 was used to visualize the vaccine-receptor complex (72), and the Blender v3.3 software was employed to render the final image (73).

### 2.8 Molecular dynamics simulation and MM-PBSA analysis

#### 2.8.1 Molecular dynamics simulation

The Molecular dynamics (MD) simulations of several complexes with better docking results were conducted using the Gromacs v2022.1 software (74), which is helpful in better understanding the dynamics and structural stability of the docking complexes. In the preprocessing stage, the Amber14SB _parmbsc1 force field parameters were added to the complex system to obtain the coordinate file and topology file of the vaccine-receptor complexes (75). Subsequently, each system was solvated using the transferable intermolecular potential 3P (TIP3P) water model, following being neutralized with Cl- ions, leaving the topological and structural coordinates in a steady state. Following this preprocessing, energy minimization was performed for the entire protein complex system; Then, under the NVT ensemble, each system was gradually heated from 0K to 310 K, and the modified Berendsen thermostat was used to equilibrium temperature (76). Then, in the NPT ensemble, the pressure of the system was adjusted to 1atm with regulating of Parinello-Rahman barostat (77). Finally, a 50ns MD simulation was run for all docking complex systems, adopting constant temperature and pressure. The LINCS algorithm (78) and the PME (Particle mesh Ewald) method (79) were used to constrain the hydrogen bond and calculate the long-range electrostatic energy, respectively. After 50ns MD simulation, the post-MD simulation analyses of the MD trajectories were performed, including the root mean square deviation (RMSD) of each system, each system chain’s root mean square fluctuation (RMSF), the radius of gyration (Rg) and hydrogen bonds of each system.

Then, the trajectories of the last 20ns were extracted for further analysis. In addition to classic MD analysis, the conformational free energy of each complex was studied at the transient stages *via* studying free energy landscapes (FELs) (80). The PCA was run using gmx covar and gmx anaeig programs, and the PC1 and PC2 were further used to calculate and analyze Gibb’s free energy landscape (Gibb’s FEL) with the gmx sham program (80). Gibb’s FEL plots were generated using the DuIvyTools (DOI:10.5281/zenodo.6340263). In Gibb’s FEL plots, the stable energy states were shown blue. In addition, the correlations between the position fluctuations of each residue in the vaccine chain and the position fluctuations of each residue in the receptor chain were analyzed with the dynamical cross-correlation (DCC) analysis by the Bio3D package in the R v4.22 software.

#### 2.8.2 MM-PBSA calculations

The Gmx_MMPBSA v1.52 tool was applied to calculate the binding free energy between the vaccine and the receptors using Poisson–Boltzmann and surface area continuum solvation (MM-PBSA) method (81), and the calculation formula is as follows:


$$\Delta G_{bind,slov}=\Delta G_{bind,vaccum}+\Delta G_{slov,complex}-\left(\Delta G_{solv,\;ligand}+\Delta G_{solv,receptor}\right)$$

### 2.9 Population coverage

More than a thousand distinct HLA alleles have been identified in humans, and the frequency of distribution of each HLA allele varies across regions and ethnicities. Given that T lymphocytes can only recognize molecular complexes composed of epitopes bound to MHC molecules, a particular epitope triggers immune responses only in individuals expressing HLA alleles with an affinity for this epitope. The IEDB population coverage tool (http://tools.iedb.org/population/) was employed to calculate the population coverage of the designed vaccine in this work based on the data of TCR specificity, HLA restriction, and HLA allele frequencies.

### 2.10 Immune simulation

For assessing the potential immune efficacy of the designed vaccine, we submitted it to the C-IMMSIM v10.1 tool (https://kraken.iac.rm.cnr.it/C-IMMSIM/) (82). The C-IMMSIM server recognizes epitopes by Position Specific Scoring Matrix (PSSM) methods, and can simultaneously stimulate the immune response of three compartments that represent three separate anatomical regions found in mammals (the bone marrow, the thymus and lymph nodes) to antigen epitopes. During the immune simulation, all parameters except for time steps were left at their default settings. In this study, the simulation time steps were set to 1050 (1-time step equals 8 h), and each injection point was set at time steps 1, 84, and 168, respectively. The default dose of each antigen injection was 1,000, and the time interval between each dose was four weeks, which is the recommended interval between injections for most commercial vaccines. To comparatively evaluate the candidate vaccine’s immunogenicity, a novel multi-epitope vaccine against Echinococcus granulosus, whose antigenicity and immunogenicity had been verified *in vitro* and *in vivo* experiments (83), was chosen as a positive control.

### 2.11 Codon optimization and *in silico* cloning

**Figure 1:**
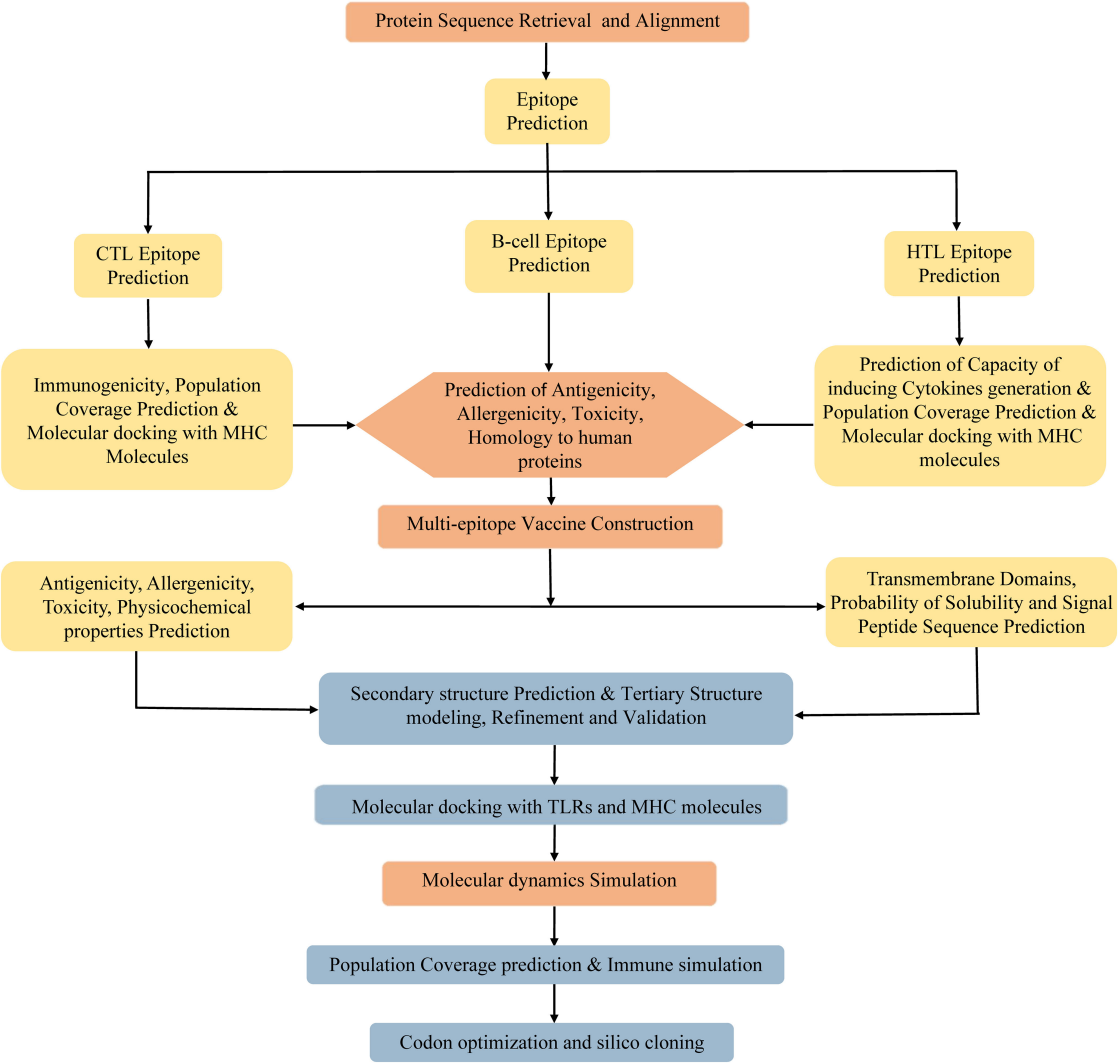
Flowchart of the multi-epitope vaccine design.

Due to the degeneracy of codons, each amino acid may correspond to multiple codons. However, there are great differences in codon preference among different species and organisms. To acquire the codon that is capable of efficiently encoding the targeted amino acid in the selected expression host, the codon optimization of the vaccine was conducted using the JCat server (http://www.jcat.de/CAICalculation.jsp) (84). The JCat server is an easy-to-use program, which can perform real-time calculations, eliminate the need for the definition of highly expressed genes, and avoid cleavage sites for specific restriction enzymes (84). The Escherichia coli (*E. coli*) K12 strain was selected as the expression host. Two parameters, codon adaptation index (CAI), and the percentage of GC content, were used to evaluate the expression efficiency of the vaccine in E. coli. The CAI, with a value range of 0 to 1, refers to the coincidence degree between synonymous codons and optimal codons, and a higher CAI indicates a higher expression level of the foreign gene in the host. The percentage of GC content represents the guanine (G)+cytosine (C) ratio in the DNA sequence, and its optimal range is 30%–70%. Otherwise, undesirable gene expression levels and transcriptional efficiency will result. Afterward, the BamHI and XhoI restriction endonuclease sites were integrated into the N- and C-terminal sites of optimized codon sequences, respectively. The entire sequence was cloned into the pET28a (+) vector using SnapGene software. Finally, the MPXV-5 vaccine protein was synthesized by Sangon Biotech co ltd (Shanghai, China). The flowchart of vaccine design is illustrated in **Figure 1**.

## 3 Result

### 3.1 Protein sequences retrieval and alignment

**Figure 2:**
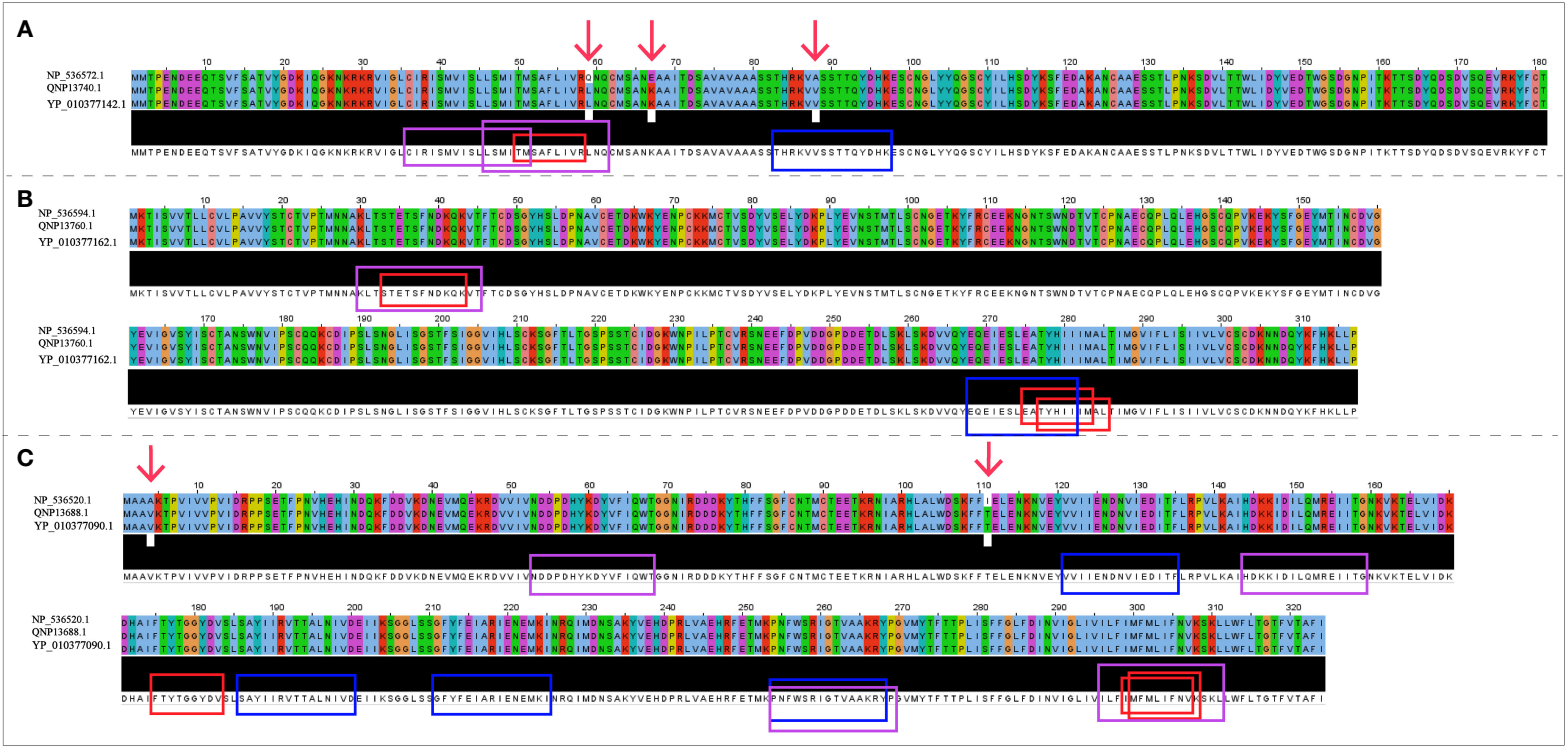
The sequence alignment of three target proteins in three selected reference strains. **(A)** The sequences of A35R; **(B)** The sequences of B6R; **(C)** The sequences of H3L; The Zaire-96-I-16 strain is selected as the reference sequence in the sequence alignment, i.e., the first-row sequences were the sequences of Zaire-96-I-16 strain, the second-row sequences were the sequences of MPXV-UK_P2 strain, and the third-row sequences were the sequences of 2022 reference strain. The red arrows refer to mutation sites; The CTL, HTL, and B cell epitopes were highlighted with red, blue and purple box lines in the sequence, respectively.

The NCBI accession numbers and amino acid sequences of A35R, B6R and H3L derived from three objective MPXV strains were presented in **Supplementary Table 1**. Protein sequence alignment results (**Figure 2**) demonstrated that there was no difference in three target protein sequences (A35R, B6R and H3L) between MPXV-UK_P2 and 2022 reference strain. Compared with the Zaire-96-I-16 MPXV strain, there were 3 mutation sites in A35R and 2 mutation sites in H3L, but no mutation sites in B6R. As shown in **Figure 2**, there were two mutation sites falling into the epitope regions.

### 3.2 Epitopes selection

**Figure 3:**
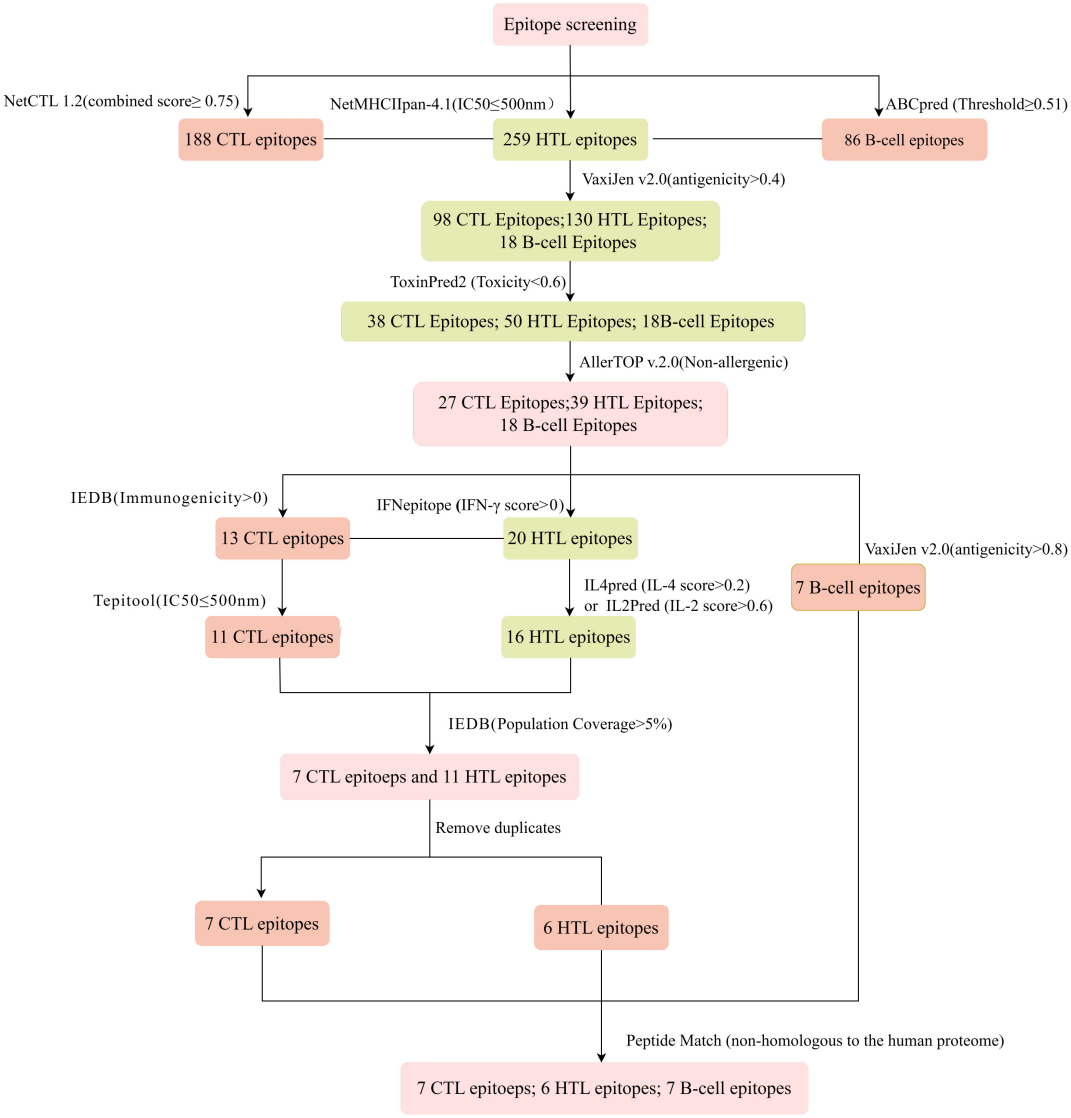
Flowchart of the epitope screening.

The immune system is comprised of many specialized cell types, all of which work together to keep people healthy. CTLs, the main force of cellular immunity, are immune cells that can directly mediate the death of target cells and eliminate circulating antigens in the host body (85). Stimulated B cells can differentiate into antibody-producing plasma cells that play a vital role in the generation of humoral immune responses (86). HTLs, another group of immune cells, can assist both humoral and cellular immunity by secreting different cytokines (87). Hence, to construct an effective multi-epitope vaccine, both T-cell epitopes (CTL and HTL epitopes) and B-cell epitopes should be incorporated. After the layer-by-layer screening (**Figure 3**), a total of 7 CTL epitopes (**Table 1**), 6 HTL epitopes (**Table 2**), and 7 liner B-cell epitopes (**Table 3**) were finally included in the vaccine constructs. The binding affinity (IC50) of the experimentally determined poxvirus-specific T cell epitopes to HLA molecules was shown in **Supplementary Table 2**.

**Table 1:** List of the CTL epitopes selected for vaccine construction.

Protein	Start position	Epitope	Antigenicity	Immunogenicity	Allergenicity	Toxicity	HLA-I alleles	IC50	Population coverage	Homology to human proteins
A35	50	TMSAFLIVR	0.5770	0.22567	NA	NT	HLA-A*31:01	20.01	52.29%	NH
							HLA-A*68:01	22.41		
							HLA-A*33:03	32.24		
							HLA-A*33:01	54.26		
							HLA-B*08:01	81.41		
							HLA-A*11:01	105.3		
							HLA-A*74:01	192.2		
							HLA-A*03:01	371.7		
B6R	275	EATYHIIIM	0.4046	0.35574	NA	NT	HLA-C*16:01	174.96	24.49%	NH
							HLA-C*12:03	239.53		
							HLA-C*03:02	370.67		
							HLA-B*35:01	410.23		
	277	TYHIIIMAL	0.4999	0.27396	NA	NT	HLA-C*14:02	14.31	44.02%	NH
							HLA-A*23:01	148.36		
							HLA-A*24:02	329.44		
							HLA-C*07:02	484.42		
	33	STETSFNDK	1.4414	0.02816	NA	NT	HLA-A*11:01	125.15	15.53%	NH
H3L	175	FTYTGGYDV	0.5030	0.1126	NA	NT	HLA-A*68:02	15.26	73.10%	NH
							HLA-A*02:06	20.57		
							HLA-C*12:03	28.93		
							HLA-C*15:02	68.73		
							HLA-C*03:02	70.97		
							HLA-C*03:03	76.09		
							HLA-C*03:04	76.09		
							HLA-C*16:01	76.1		
							HLA-A*02:01	143.89		
							HLA-C*17:01	159.26		
							HLA-C*12:02	217.08		
							HLA-C*02:02	328.68		
							HLA-C*02:09	328.68		
	299	IMFMLIFNV	0.4464	0.0708	NA	NT	HLA-A*02:01	6.73	44.14%	NH
							HLA-A*02:06	15.39		
							HLA-A*32:01	322.73		
	300	MFMLIFNVK	1.1933	0.1903	NA	NT	HLA-A*33:01	105.2	20.7%	NH
							HLA-A*31:01	145.5		
							HLA-A*68:01	157.1		
							HLA-A*33:03	180.6		
							HLA-A*30:01	261.4		

NT, Non-toxic; NA, Non-allergenic; NH, Non-homologous to human proteins.

**Table 2:** List of the HTL epitopes selected for vaccine construction.

Protein	Start position	Epitope	HLA-II alleles	IC50	Rank%	Antigenicity	Allergenicity	Toxicity	IFN-γ score	IL-4 score	IL-2 score	Population coverage	Homology to human proteins
A35	83	THRKVVSSTTQYDHK	HLA-DRB1_0405	141.45	0.29	0.7	NA	NT	0.29103344	0.34	0.591	24.24%	NH
			HLA-DRB1_0404	323.42	1.77								
			HLA-DRB1_0401	367.21	2.61								
			HLA-DRB1_0901	393.12	1.08								
B6R	268	EQEIESLEATYHIII	HLA-DRB1_0101	46.96	1.82	0.7	NA	NT	0.95981859	1.41	0.662	11.53%	NH
H3L	254	PNFWSRIGTVAAKRY	HLA-DRB1_0101	11.51	2.5	1.1	NA	NT	0.092049799	- 0.11	0.542	37.70%	NH
			HLA-DRB1_0401	26.99	0.34								
			HLA-DRB1_1101	60.33	3.18								
			HLA-DRB1_0405	68.39	1.99								
			HLA-DRB1_0404	77.11	3.37								
			HLA-DRB3_0202	428.23	4.72								
	186	SAYIIRVTTALNIVD	HLA-DRB1_0701	19.1	0.53	0.8	NA	NT	0.14153398	0.67	0.536	40.98%	NH
			HLA-DRB1_0404	29.89	1.61								
			HLA-DRB1_1501	68.69	4.39								
			HLA-DRB1_0405	69.85	4.88								
	121	VVIIENDNVIEDITF	HLA-DRB3_0101	185.04	2.68	0.6	NA	NT	0.70710024	0.31	0.602	30.81%	NH
			HLA-DRB1_0301	278.97	2.42								
			HLA-DRB3_0202	406.75	4.41								
			HLA-DRB1_0405	410.33	3.45								
	211	GFYFEIARIENEMKI	HLA-DRB1_0405	49.22	2.17	0.6	NA	NT	0.0688676505	1.36	0.478	19.34%	NH
			HLA-DRB1_1101	67.46	2.36								
			HLA-DRB1_0901	111.2	3.62								

NT, Non-toxic; NA, Non-allergenic; Epitopes with positive IFN-γ scores, predicted using SVM model, are graded as IFN-γ inducers; Epitopes with an IL-4 score >0.2 are graded as IL-4 inducers; Epitopes with an IL-2 score >0.6 are graded as IL-2 inducers; NH, Non-homologous to human proteins.

**Table 3:** List of the B-cell epitopes selected for vaccine construction.

Protein	Start position	Epitope	Antigenicity	Toxicity	Allergenicity	Homology to human proteins
A35	36	CIRISMVISLLSMITM	1.1317	NT	NA	NH
	46	LSMITMSAFLIVRQNQ^a^	0.8507	NT	NA	NH
B6R	30	KLTSTETSFNDKQKVT	1.1997	NT	NA	NH
H3L	53	NDDPDHYKDYVFIQWT	0.9084	NT	NA	NH
	144	HDKKIDILQMREIITG	0.8853	NT	NA	NH
	254	PNFWSRIGTVAAKRYP	1.0417	NT	NA	NH
	296	ILFIMFMLIFNVKSKL	1.2217	NT	NA	NH

Epitope^a^: The epitope only appears in the Zaire-96-I-16 strain. NT, Non-toxic; NA, Non-allergenic; NH, Non-homologous to human proteins.

### 3.3 Molecular docking of the T-cell epitopes with MHC molecules

Except for only one epitope (STETSFNDK), all other epitopes had multiple binding alleles. Some had even as high as 8 (TMSAFLIVR) or 13 (FTYTGGYDV) alleles (**Table 1**). In this study, we performed molecular docking between each T-cell epitope and one of their corresponding alleles for a representative docking analysis (**Supplementary Table 3**). As shown in **Supplementary Table 3**, Autodock Vina docking data indicated that the binding energy between CTL epitopes and MHC class I alleles ranged from -10.2 to -5.9 Kcal/mol, and the binding energy range between HTL epitopes and MHC class II alleles was -7 to -5.8 Kcal/mol, the binding energy range between seven experimental verified antigen epitopes of poxvirus and HLA-A*02:01 molecules was -9 to -5.8 Kcal/mol, indicating the T-cell epitopes had a good affinity for MHC molecules. The predicted results of Ligplus v2.25 demonstrated that many hydrogen bonds were formed between T-cell epitopes and MHC molecules, and salt bridges were also detected in certain docking cases (**Supplementary Table 3**), which once again proved that T-cell epitopes had a strong affinity to MHC molecules.

### 3.4 Multi-epitope vaccine features

#### 3.4.1 Prediction of antigenicity, allergenicity, and toxicity of the vaccine construct

The final multi-epitope vaccine construct was built with the following six parts: CTL epitopes, HTL epitopes, B-cell epitopes, PADRE, adjuvant and His-tag sequence. Five different adjuvants (50S ribosomal protein L7/L12, β-defensin, LT-IIC, CTB, RS09) resulted in five different vaccine constructs (**Supplementary Table 4**) named MPXV-1, MPXV-2, MPXV-3, MPXV-4 and MPXV-5, respectively. Except for the adjuvant, all other components of these constructs are identical. The antigenicity ranges of five vaccine constructs predicted by VaxiJen and ANTIGENpro servers were 0.62~0.69 and 0.56~0.84, respectively, which demonstrated that the five vaccine constructs can bind or interact with the immune effector cells or soluble antibodies (**Table 4**). Moreover, none of the five vaccine constructs were allergenic. However, the ToxinPred2 predicted results showed that MPXV-3 and MPXV-4 were toxic. So, the vaccine bodies of MPXV-3-4 and their corresponding adjuvants were separately submitted to the ToxinPred2 server, and its results revealed that the vaccine bodies were not toxic, while both adjuvants were toxic.

**Table 4:** Properties of the vaccine constructs.

Properties	V1	V2	V3	V4	V5
adjuvant	50S ribosomal L7/L12	Beta-defensin	LT-IIC	Cholera Toxin B subunit	RS09
				(CTB)	
Antigenicity (Vaxijen/ANTIGENpro)	0.62/0.56	0.68/0.60	0.64/0.59	0.66/0.84	0.69/0.59
Allergenicity (AllerTOP v2.0+AlgPred)	NA	NA	NA	NA	NA
Toxicity	NT	NT	toxic	toxic	NT
Number of amino acids	511	426	479	484	388
Molecular weight (Da)	56700.70	48421.41	53920.37	54905.58	43952.00
Theoretical isoelectric point (pI)	8.61	9.56	9.35	9.27	9.39
The estimated half-life^(a)^	30 hours	30 hours	30 hours	30 hours	30 hours
The estimated half-life^(b)^	>20 hours	>20 hours	>20 hours	>20 hours	>20 hours
The estimated half-life^(c)^	>10 hours	>10 hours	>10 hours	>10 hours	>10 hours
The instability index (II)	30.91	36.62	34.19	35.65	35.68
Aliphatic index	89.06	82.25	84.15	84.52	84.05
Grand average of hydropathicity (GRAVY)	-0.051	-0.19	-0.13	-0.171	-0.127
Solubility	0.975118	0.835770	0.610239	0.867569	0.869869

The estimated half-life^(a)^: Estimated half-life of the vaccine in mammalian reticulocytes (*in vitro*); The estimated half-life^(b)^: Estimated half-life of the vaccine in yeast (*in vitro*); The estimated half-life^(c)^: Estimated half-life of the vaccine in *Escherichia coli (in vivo)*.

NA, Non-allergenic; NT, Non-toxic.

#### 3.4.2 Physicochemical properties analysis of the vaccine construct

The physicochemical properties of the vaccine constructs are presented in **Table 4**. The molecular weight of five vaccine constructs (MPXV-1–5) was below 110 kDa, which was considered favorable for vaccine development. The theoretical pI for all vaccine constructs ranged from 8.61 to 9.56. The range of instability index (II) values for all constructs was between 34.19 and 36.62, suggesting that the constructs were stable in a test tube. In general, proteins with an instability index of less than 40 were considered to be stable. The aliphatic index is regarded as a positive factor for the increase in thermostability of globular proteins. In all developed constructs, the aliphatic index ranged from 82.25 to 89.06, indicating that these constructs were thermostable. All designed vaccines have an estimated half-life of 30 hours in mammalian reticulocytes, >20 hours in yeast, and >10 hours in E. coli. The range of the GRAVY index of MPXV-1-5 was -0.19 to -0.051. The GRAVY index is correlated with protein solubility, with negative GRAVY indicating a hydrophilic molecule.

Predictions from the DeepTMHMM server showed that none of the constructed vaccines, except MPXV-1, has a transmembrane helix (**Supplementary Figure 1**). This may be due to the adjuvant of MPXV-1 is located inside the membrane while the vaccine body is located outside the membrane, resulting in the formation of transmembrane sequences at the bridging site between the two parts. The probabilities of being soluble upon overexpression in E. coli of five constructs were 0.975118 (MPXV-1), 0.835770 (MPXV-2), 0.610239 (MPXV-3), and 0.867569 (MPXV-4), 0.869869 (MPXV-5), respectively, which demonstrated that all vaccine constructs had good solubility when heterologous expression in E. coli. Moreover, the predicted results of the signalP-6.0 server revealed that none of the vaccines had signal peptide sequences (**Supplementary Figure 2**).

Based on all the above predictions about the properties of all vaccine constructs, MPXV-2 and MPXV-5 were regarded as the most ideal vaccine constructs for immunological applications. Therefore, MPXV-2 and MPXV-5 were selected for further analysis.

### 3.5 Secondary structure prediction of the vaccine construct

The secondary structures of the final selected vaccine constructs were predicted by the PSIPRED server and predicted results (**Supplementary Figure 3**) showed that the MPXV-2 contained 58.22% α-helix, 31.22% coils and 10.56% β-strands. Compared to MPXV-2, the MPXV-5 secondary structure comprised a greater proportion of α-helix (61.10%), a smaller proportion of β-strands (6.79%), with the similar proportion of coils (32.11%) (**Supplementary Figure 3**).

### 3.6 Three-dimensional structure prediction, refinement, and validation of the vaccine construct

**Figure 4:**
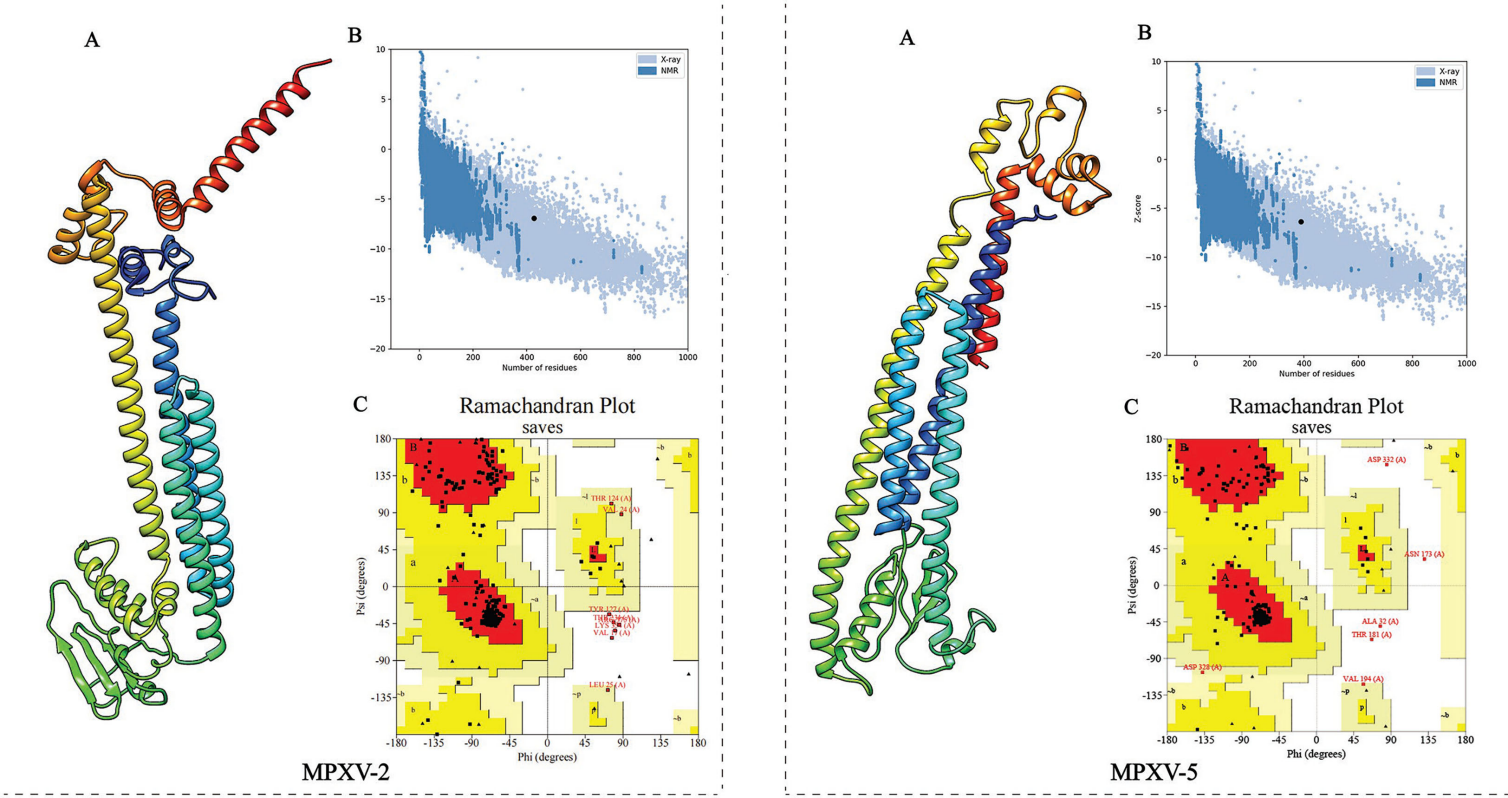
Refinement and quality validation of the three dimensional (3D) structure. **(A)** The refined 3D structure. **(C)** Ramachandran plots of the refined 3D structure generated by PROCHECK server; Red regions represent the most favored regions, dark yellow regions represent the additional allowed region, light yellow regions represent the generally allowed regions, and white regions denote the disallowed regions. **(B)** Z-Score plot of the refined 3D structure predicted by ProSA-Web server.

The 3D structure of the selected vaccine constructs was predicted by the Robetta server and then refined by the GalaxyWEB server. Based on the model quality of the refined models (**Supplementary Table 5**), the refined model 5 (**Figure 4A**) for MPXV-2 and the refined model 4 (**Figure 4A**) for MPXV-5 were chosen for further quality validation. The corresponding Overall Quality Factors generated by the ERRAT program were 91.8465 (MPXV-2-5) and 89.2105 (MPXV-5-4) (**Supplementary Figure 4**), and the corresponding Z-scores predicted by the ProSA-Web server were -6.94 (MPXV-2-5) and -6.36 (MPXV-5-4) (**Figure 4B**). The Ramachandran plot analysis of the refined model MPXV-2-5 revealed that 92.3% of residues located in the most favorable region, 6.4% in the allowed region, and only 1.3% in the disallowed region (**Figure 4C**). For the refined model MPXV-5-4, 92.4% of residues suited in the most favored region, 6.4% in the allowed region, and only 1.2% of residues in the disallowed regions (**Figure 4C**). The above analysis confirmed the excellent quality of the refined 3D models of the MPXV-2 and MPXV-5 vaccine constructs.

### 3.7 Molecular docking

**Figure 5:**
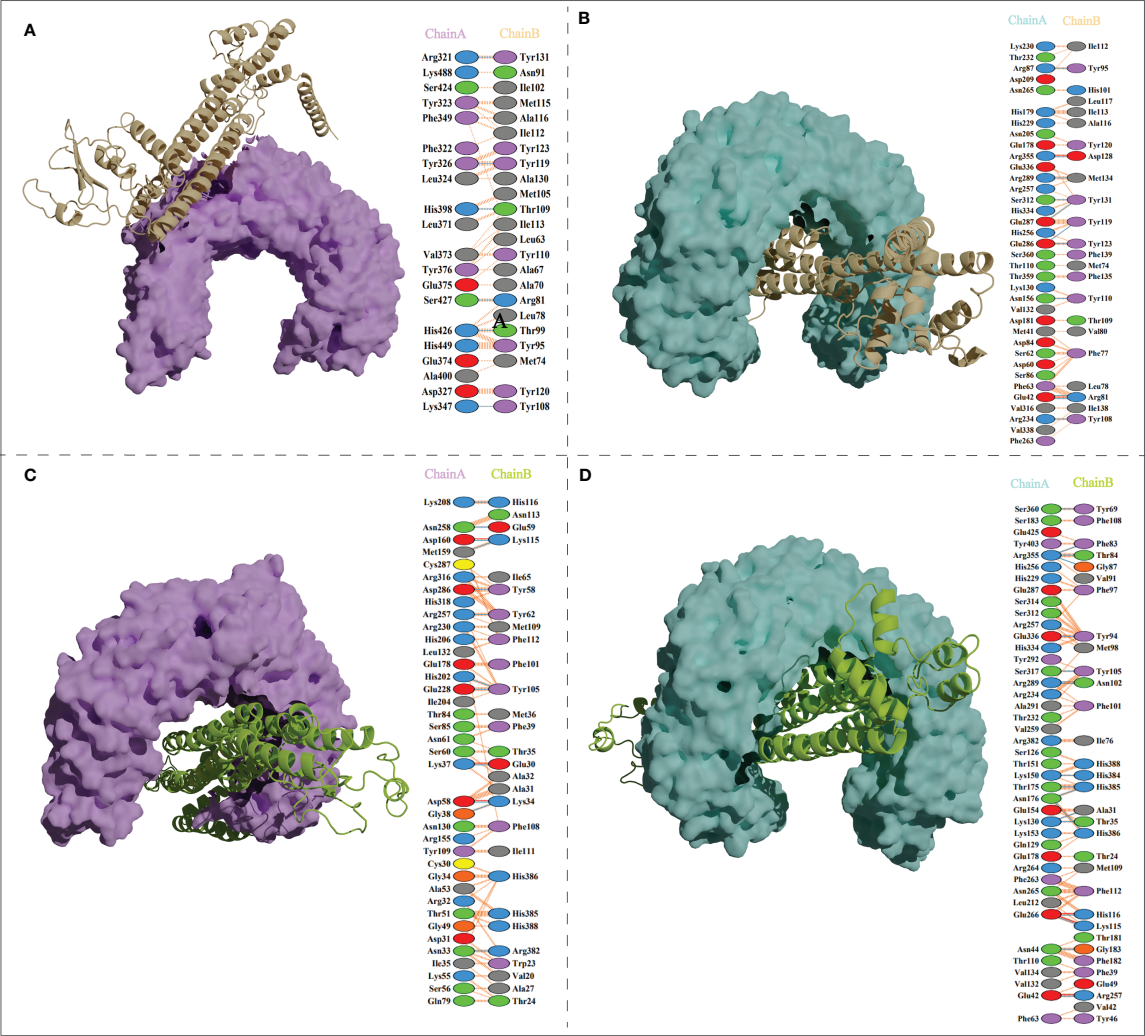
Vaccine candidates (MPXV-2 and MPXV-5) docked with TLR2 and TLR4. **(A)** The binding mode between the MPXV-2 and TLR2 and the interacting residues of the MPXV-2 with TLR2; **(B)**The binding mode between the MPXV-2 and TLR4 and the interacting residues of the MPXV-2 with TLR4; **(C)** The binding mode between the MPXV-5 and TLR2 and the interacting residues of the MPXV-5 with TLR2; **(D)** The binding mode between the MPXV-5 and TLR4 and the interacting residues of the MPXV-5 with TLR4.

The ClusPro generated thirty clusters in each docking case, and the best one with the largest cluster size in each docking case was selected to present (**Table 5**). **Table 5** displayed the detailed docking results for all refined docking complexes. Compared with the MPXV-2-immune receptor complexes, all corresponding MPXV-5-immune receptor complexes had lower docking scores and binding energy, indicating that MPXV-5 had stronger affinity for all immune receptors (TLR2, TLR4, HLA-A*02:01 and HLA-DRB1*01:01). The predicted results of the PDBsum server (**Figure 5**; **Supplementary Figure 5** and **Table 5**) showed that 6 hydrogen bonds, 11 hydrogen bonds and 2 salt bridges, 12 hydrogen bonds and 1 salt bridges, 16 hydrogen bonds and 6 salt bridges were formed in MPXV-2-TLR2, MPXV-2-TLR4, MPXV-2-HLA-A*02:01 and MPXV-2-HLA-DRB1*02:01 complexes, respectively. There are 12 hydrogen bonds with 3 salt bridges, 18 hydrogen bonds with 3 salt bridges, 12 hydrogen bonds with 3 salt bridges, and 12 hydrogen bonds with 5 salt bridges in MPXV-5-TLR2, MPXV-5-TLR4 and MPXV-5-HLA-A*02:01 and MPXV-HLA-DRB1*02:01 complexes, respectively (**Figure 5**; **Supplementary Figure 5** and **Table 5**). Docking studies showed that there were significant interactions between the designed vaccines (MPXV-2 and MPXV-5) and the immune receptors.

**Table 5:** Docking analysis of vaccine-receptors complexes.

Server	Parameter	MPXV-2-TLR2	MPXV-2-TLR4	MPXV-2-HLA-A*02:01	MPXV-2-HLA-DRB1*01:01	MPXV-5-TLR2	MPXV-5-TLR4	MPXV-5-HLA-A*02:01	MPXV-5-HLA-DRB1*01:01
ClusPro 2.0	Center	-953.1	-1088	-998.6	-1092.7	-1203.8	-1166.8	-875.8	-1224.5
	Lowest Energy	-1110.3	-1473.3	-1033.6	-1330.8	-1203.8	-1261.8	-1037.5	-1224.5
HADDOCK 2.4	HADDOCK score	-171.0 +/- 0.9	-242.1 +/- 3.7	-378.0 +/- 3.4	-676.9 +/- 2.4	-275.6 +/- 3.2	-306.2 +/- 1.6	-423.8 +/- 7.2	-675.7 +/- 1.7
	Cluster size	20	20	20	20	20	20	20	20
	RMSD from the overall lowest-energy structure	0.6 +/- 0.4	0.6 +/- 0.3	0.6 +/- 0.3	0.6 +/- 0.3	0.6 +/- 0.4	0.6 +/- 0.3	0.6 +/- 0.3	0.6 +/- 0.3
	Van der Waals energy	-84.8 +/- 2.3	-115.0 +/- 2.6	-176.2 +/- 2.2	-304.9 +/- 6.4	-122.2 +/- 1.4	-138.5 +/- 1.8	-186.7 +/- 3.0	-316.1 +/- 2.4
	Electrostatic energy	-108.1 +/- 6.0	-297.5 +/- 22.0	-589.8 +/- 25.1	-1254.2 +/- 23.7	-364.6 +/- 11.1	-323.4 +/- 35.9	-703.2 +/- 17.4	-1154.4 +/- 21.3
	Desolvation energy	-64.6 +/- 3.3	-67.6 +/- 2.2	-83.7 +/- 2.0	-121.1 +/- 1.4	-80.4 +/- 1.4	-103.0 +/- 5.7	-96.5 +/- 6.8	-128.8 +/- 4.2
	Restraints violation energy	0.0 +/- 0.0	0.0 +/- 0.0	0.0 +/- 0.0	0.0 +/- 0.0	0.0 +/- 0.0	0.0 +/- 0.0	0.0 +/- 0.0	0.0 +/- 0.0
	Buried Surface Area	2557.1 +/- 16.1	3619.9 +/- 65.0	5011.4 +/- 65.8	7923.8 +/- 34.6	3871.2 +/- 70.5	4361.5 +/- 75.1	5447.7 +/- 98.5	8418.8 +/- 83.3
	Z-Score	0	0	0	0	0	0	0	0
PRODIG	ΔG (kcal/mol)	-9.4	-19.1	-11.9	-11.6	-16.1	-16.5	-12.9	-12.3
	Kd (M) at 25.0 °C	1.30E-07	1.00E-14	1.80E-09	2.90E-09	1.70E-12	7.70E-13	3.50E-10	8.80E-10
PDBsum	Number of hydrogen bonds	6	11	12	16	12	18	12	12
	Number of salt Bridges	0	2	1	6	3	3	3	5

ΔG, binding energy; Kd, dissociation constant.

### 3.8 Molecular dynamics simulation

#### 3.8.1 Root mean square deviation, root mean square fluctuation radius of gyration and hydrogen bonds analyses

**Figure 6:**
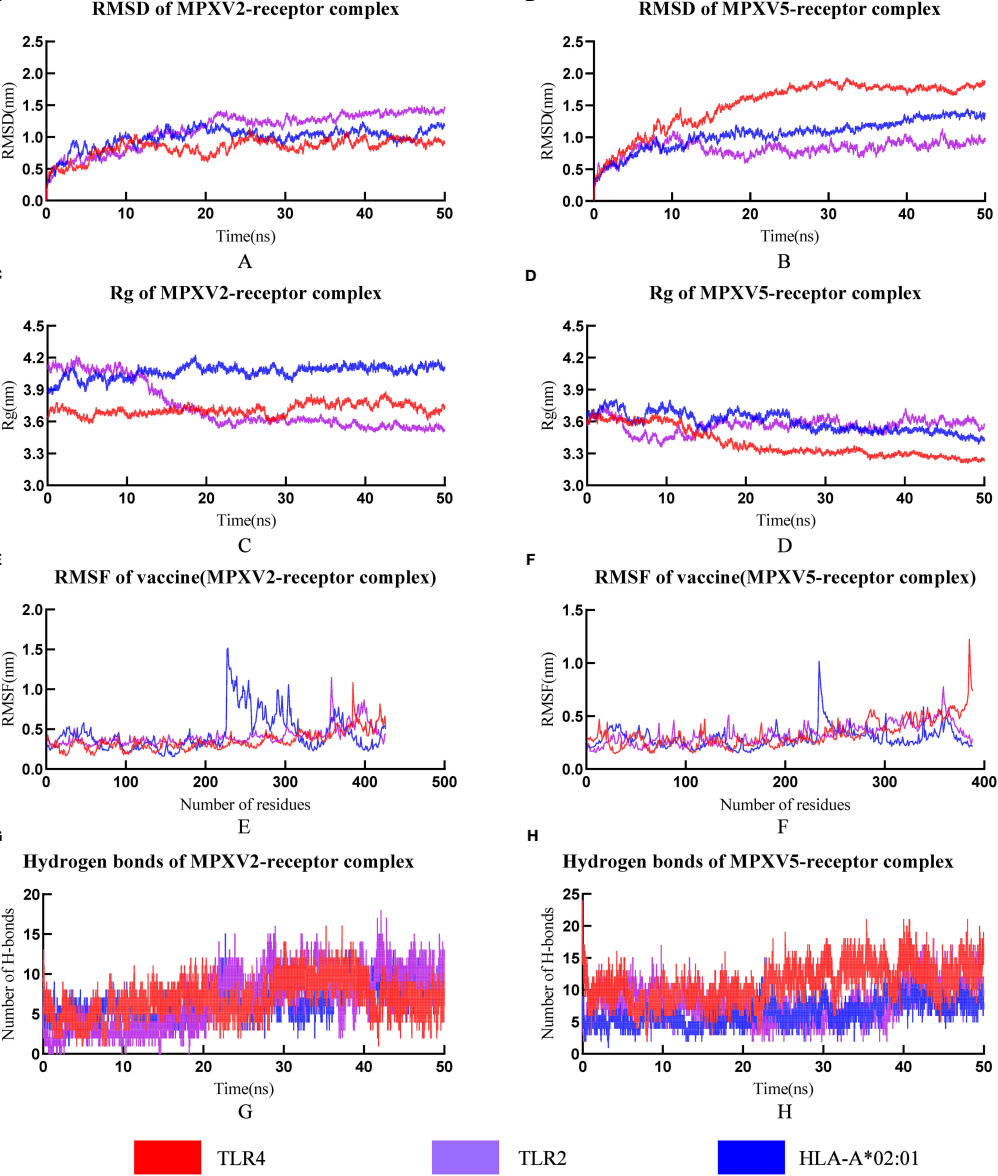
The plot of Root means square deviation (RMSD), Root means square fluctuation (RMSF), Radius of Gyration (Rg) and hydrogen bonds of vaccine-receptor complexes during the Molecular dynamics (MD) simulation. **(A)** RMSD of MPXV-2-TLR2, MPXV-2-TLR4 and MPXV-2-HLA-A*02:01 complexes; **(B)** RMSD of MPXV-5-TLR2, MPXV-5-TLR4 and MPXV-5-HLA-A*02:01 complexes; **(C)** Rg of MPXV-2-TLR2, MPXV-2-TLR4 and MPXV-2-HLA-A*02:01 complexes; **(D)** Rg of MPXV-5-TLR2, MPXV-5-TLR4 and MPXV-5-HLA-A*02:01 complexes; **(E)** RMSF of MPXV-2 chain in MPXV-5-TLR2, MPXV-5-TLR4 and MPXV-5-HLA-A*02:01 complexes; **(F)** RMSF of MPXV-5 chain in MPXV-5-TLR2, MPXV-5-TLR4 and MPXV-5-HLA-A*02:01; **(G)** Hydrogen bonds in MPXV-2-TLR2, MPXV-2-TLR4 and MPXV-2-HLA-A*02:01 complexes; **(H)** Hydrogen bonds in MPXV-5-TLR2, MPXV-5-TLR4 and MPXV-5-HLA-A*02:01 complexes.

RMSD can be used to measure changes in protein structure during MD simulations compared to the starting point. A leveling off of the RMSD curve can also indicate that the protein structure has equilibrated. The RMSD value of all MPXV-2-receptors complexes (MPXV-2-TLR2, MPXV-2-TLR4, MPXV-2-HLA-A*01:01) increased gradually in the range of 1-20ns and then maintained the level of about 1.3nm, 0.9nm, 1.0nm, respectively (**Figure 6A**). The average RMSD value of the MPXV-5-TLR2 was 0.82nm (**Figure 6B**), and several small fluctuations in RMSD value were observed. The average RMSD value of MPXV-5-TLR4 complex was the highest among all complexes, which was 1.51nm (**Figure 6B**). The RMSD value of the MPXV-5-HLA-A*01:01 complex stabilized at about 20 ns of the MD simulation with an average value of 1.05 nm (**Figure 6B**).

The radius of gyration (Rg) is defined as the distribution of atoms of a protein around its axis and can be used to characterize the compactness of molecules. To some extent, it can characterize the overall changes in the ligand structure upon binding to the receptor protein. In this study, the RG values of MPXV-2-TLR4 and MPXV-2-HLA-A*02:01 complex systems were all kept at a relatively constant level (**Figure 6C**), indicating that the protein complex system was relatively stable with no obvious conformational change. The RG value of the MPXV-2-TLR2 complex initially maintained a stable level, began to decline at about 9 ns and reached a stable level again at about 22 ns (**Figure 6C**). The RG values of all MPXV-5-receptors complexes (MPXV-5-TLR2, MPXV-5-TLR4, MPXV-5-HLA-A*01:01) fluctuated slightly in the initial phase, and keep stable at about 20ns of the MD simulation (**Figure 6D**).

RMSF is a measurement of individual residue flexibility and the higher the RMSF value indicates better flexibility. The RMSF value of MPXV-2 chain in complex with TLR2 and TLR4 had less magnitude of fluctuation with an average value of 0.40 ± 0.11nm and 0.35 ± 0.12 nm, respectively (**Figure 6E**). In the case of MPXV-2 chain complexed with HLA-A*02:01, the RMSF value of residues 228 to 317 showed significant fluctuations, reaching a peak value of 1.51nm (**Figure 6E**). The RMSF value of MPXV-5 chain in complex with TLR2 had little magnitude of fluctuation with an average value of 0.33 ± 0.09 nm (**Figure 6F**). In the case of MPXV-5 chain in the MPXV-5-TLR4 and MPXV-5-HLA-A-02:01 complexes, residues 386-389 and residues 235-241 showed the largest magnitude of fluctuations, respectively, and the average RMSF value of them was 0.33 ± 0.13nm and 0.29 ± 0.09 nm, respectively (**Figure 6F**). For MPXV-2-receptors complexes, the RMSF values of almost all residues of the TLR2 chain were less than 0.5 nm, except for a few residues, such as residues 34-36 and 377-381(**Supplementary Figure 6**); for the TLR4 chain, the maximum and minimum RMSF values were 0.5838 nm and 0.1759 nm, respectively, and the RMSF values of bulk amino acids were less than 0.5nm (**Supplementary Figure 6**); for both A chain and B chain of HLA-A-02:01, the RMSF values showed major fluctuations in almost all residues. For MPXV-5-receptor complexes, the RMSF values of the TLR2 chain and HLA-A*02:01 chain had less magnitude of fluctuation, with an average value of 0.24nm ± 0.06 and 0.24 ± 0.05/0.30 ± 0.07(A chain/B chain), respectively (**Supplementary Figure 6**). In the MPXV-5-TLR4 complex, the maximum and minimum RMSF values in the TLR4 chain were 0.66 and 0,16, with a higher average value of 0.32 ± 0.09 nm (**Supplementary Figure 6**).

Hydrogen bonds is a nonbonded interaction, which is essential in maintaining the overall stability of the vaccine-receptor complex. In general, the number of hydrogen bonds in all complexes except the MPXV-5-TLR4 complex increased with time. The number of hydrogen bonds in MPXV-2-TLR2, MPXV-2-HLA-A02:01, MPXV-2-TLR4, MPXV-5-TLR2, MPXV-5-HLA-A02:01, MPXV-5-TLR4 complexes finally stabilized at approximately 7, 12, 8, 11,15 and 8, respectively (**Figures 6G, H**). The calculations of RMSD, RG, RMSF and hydrogen bonds demonstrated the stability and stiffness of the vaccine-TLRs and vaccine-HLA-A-02:01 complexes.

#### 3.8.2 Gibb’s free energy analysis

**Figure 7:**
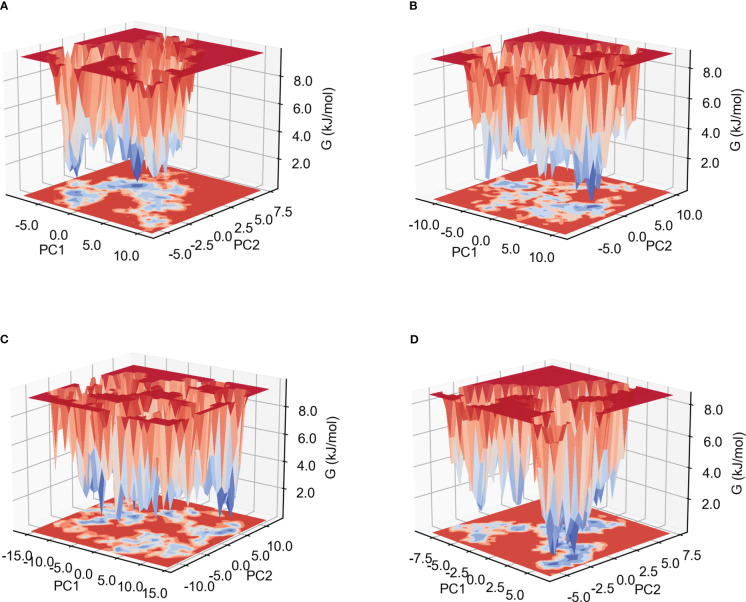
Gibb’s free energy landscape. **(A)** MPXV-2-TLR2 complex. **(B)** MPXV-5-TLR2 complex. **(C)** MPXV-2-TLR4 complex. **(D)** MPXV-5-TLR4 complex.

The low Gibb’s free energy value corresponds to the stable conformation and system. In this study, the calculation of Gibb’s free energy value was based on the two principal components: the two-dimensional projections of PC1 *vs* PC2 were analyzed. As shown in **Figure 7** and **Supplementary Figure 7**, among the six complexes, MPXV-5-TLR4, MPXV-2-HLA-A*02:01 and MPXV-2-TLR4 complexes have more lowest energy basins, followed by MPXV-5-TLR3 and MPXV-5-HLA-A*02:01, MPXV-5-TLR2 complexes, while MPXV-5-TLR2 complex has the fewer lowest energy basins. The lowest energy conformations of the MPXV-2-TLR2 complex were limited to the narrow range of energy basins -8.7 to 10.8 kJ/mol on PC1 and -5.6 to 7.5 kJ/mol on PC2(**Figure 7A**). For the MPXV-5-TLR2 complex, the energy range for PC1 was -11.9 to 10.7 kJ/mol and -8.6 to 10.8 kJ/mol for PC2 (**Figure 7B**). For the MPXV-2-TLR4 complex, the energy range for PC1 was -16.1 to 14.1 kJ/mol and -11.6 to 12.7 kJ/mol for PC2 (**Figure 7C**). For the MPXV-5-TLR4 complex, the energy range was -8.8 to 6.6 kJ/mol for PC1 and -5.8 to 7.4 kJ/mol for PC2 (**Figure 7D**). For the MPXV-2- HLA-A*02:01 complex, the energy range was wider, with PC1 having an energy range of -16.1 to 15.2 kJ/mol and PC2 having an energy range of -11.4 to 12.7 kJ/mol (**Supplementary Figure 7A**). For the MPXV-5-HLA-A*02:01 complex, the energy range for PC1 was -8.9 to 16.0 kJ/mol and -6.2 to 8.2 kJ/mol for PC2 (**Supplementary Figure 7B**).

#### 3.8.3 MM-PBSA calculation

The trajectories extracted at 100 ps during the 40-50-ns simulation periods were submitted to MM-PBSA calculation. As shown in **Table 6**, the total binding free energies (ΔTOTAL) of MPXV-2-TLR2, MPXV-2-TLR4, MPXV-2-HLA-A*02:01, MPXV-5-TLR2, MPXV-5-TLR4, MPXV-5-HLA- A*02:01 complexes were -143.12 kcal/mol, -129.11 kcal/mol, -126.44 kcal/mol, -103.64 kcal/mol, -184.82kcal/mol, and -100.55 kcal/mol, respectively, indicating that both MPXV-2 and MPXV-5 have good affinity to TLR2, TLR4 and HLA-A*02:01. The analysis of detailed interaction energies was shown in **Table 6**.

**Table 6:** MM-PBSA calculations of vaccines-receptors complexes.

Energy component	Average (MPXV-2)			Average (MPXV-5)		
	TLR2	TLR4	HLA-A*02:01	TLR2	TLR4	HLA-A*02:01
ΔVDWAALS(KJ/mol)	-164.52	-139.06	-143.93	-147.37	-223.37	-166.71
ΔEEL(KJ/mol)	-3030.72	-3750.02	-1574.48	-792.96	-2822.84	-116.11
ΔEPB(KJ/mol)	3072.22	3777.71	1609.49	853.81	2890.32	201.12
ΔENPOLAR(KJ/mol)	-20.10	-17.73	-17.51	-17.12	-28.93	-18.85
ΔGGAS(KJ/mol)	-3195.24	-3889.09	-1718.41	-940.33	-3046.21	-282.82
ΔGSOLV(KJ/mol)	3052.13	3759.98	1591.97	836.69	2861.38	182.27
ΔTOTAL(KJ/mol)	-143.12	-129.11	-126.44	-103.64	-184.82	-100.55

ΔVDWAALS, van der Waals energy; ΔEEL, Electrostatic energies; ΔEPB, Polar solvation energy; ΔENPOLAR, Nonpolar solvation energy; ΔGGAS = ΔVDWAALS+ΔEEL; ΔGSOLV = ΔEPB+ΔENPOLAR; ΔTOTAL = ΔGSOLV +ΔGGAS.

#### 3.8.4 Dynamic cross-correlation analysis

The correlations between the movements of different residues of the two chains in the vaccine-receptor complex systems were shown with the DCCM generated by DDC analysis. In the figure of DCCM, the direction and strength of the correlation between different residues were shown in different colors, and the red, white and blue corresponded to the correlation coefficients of 1 0, and -1, respectively. As shown in **Supplementary Figure 8**, both positively and negatively correlated residue contacts were detected between all vaccine chains and all receptor chains. In the MPXV-2-TLR2 complex, the residues around 51-151 (residues 600-700 in the figure) in the vaccine chain showed a slightly positive correlation with the residues 240-500 (residues 240-500 in the figure) from the TLR2 chain (**Supplementary Figure 8A**). In the MPXV-2-TLR4 complex, the relatively strong positive correlations between the residues 49-149 and 299-339 (residues 650-750, 900-940 in the figure) from the vaccine chain and the TLR4 chain were observed (**Supplementary Figure 8B**). In the MPXV-2-HLA-A*02:01 complex, the residues 28-128 (residues 400-500 in the figure) from the vaccine chain showed a positive correlation with the residues 1-200 (residues 1-200 in the figure) from the A chain of HLA-A*02:01 (**Supplementary Figure 8C**). In the MPXV-5-TLR2 complex, the residues around 26-46, 61-111 and 251-331(residues 575-595, 610-650 and 800-880 in the figure) from the vaccine chain showed positive correlations with the residues 1-300 (residues 1-300 in the figure) from the TLR2 chain (**Supplementary Figure 8D**). In the MPXV-5-TLR4 complex, the residues around 9-109 and 249-299 (residues 610-710 and 850-900 in the figure) from the vaccine chain showed positive correlations with the residues 1-250 (residues 1-250 in the figure) from the TLR4 chain (**Supplementary Figure 8E**). In the MPXV-5-HLA-A*02:01 complex, the residues around 28-118 (residues 400-490 in the figure) from the vaccine chain showed positive correlations with the residues 1-190 (residues 1-190 in the figure) from the HLA-A*02:01 chain (**Supplementary Figure 8F**).

### 3.9 Population coverage

**Figure 8:**
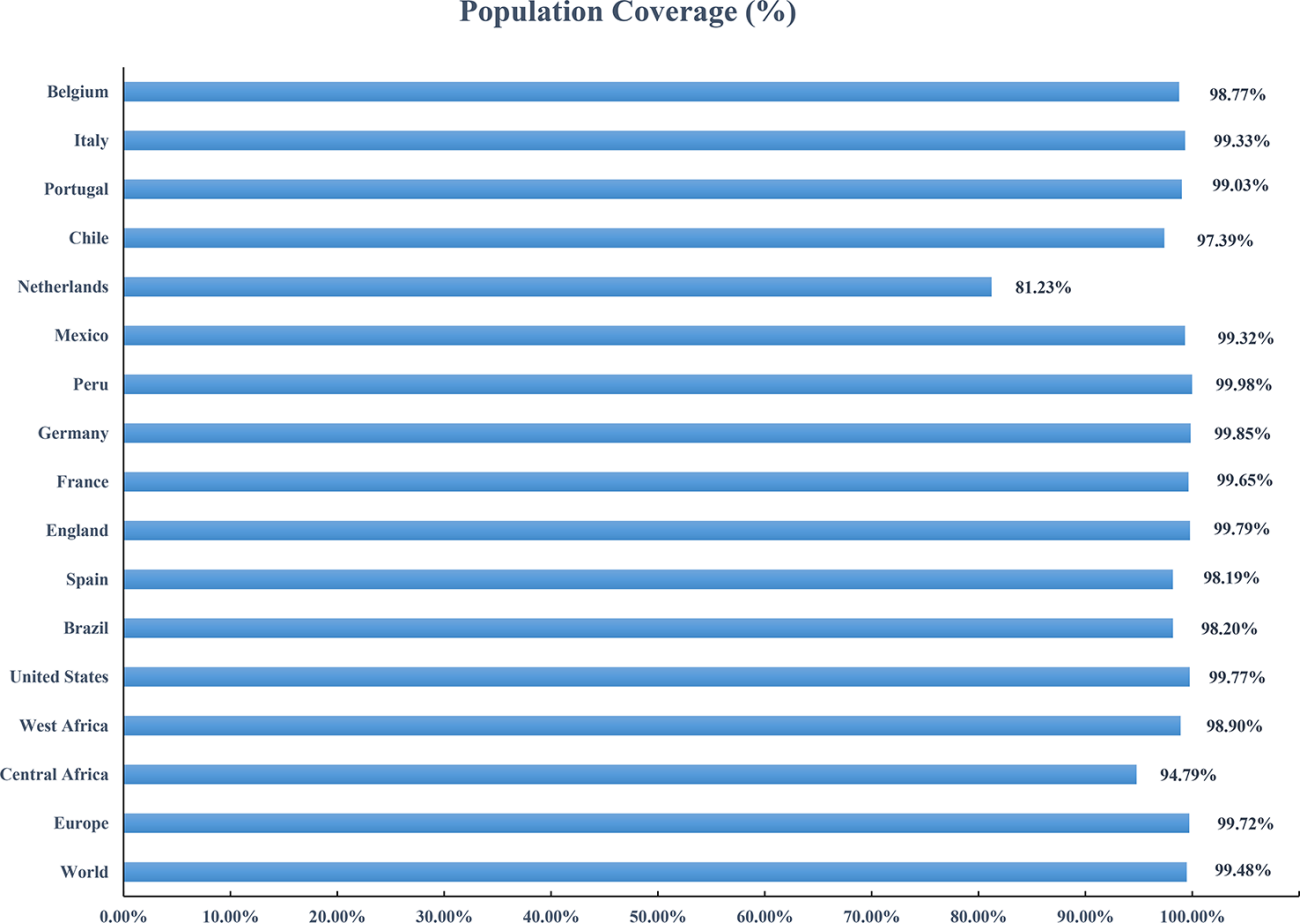
Population coverage of the designed vaccine in different regions suffering relatively severe MPXV attacks is calculated by the IEDB population coverage tool.

Based on the HLA alleles binding to T-cell epitopes in the multi-epitope vaccine, the population coverage predictions were performed to determine the proportion of the population in different parts of the world that would respond to the designed vaccines. As shown in **Figure 8**, the population coverage of the multi-epitope vaccine was high in countries suffering relatively severe MPXV attacks in this outbreak, such as Europe (Spain, France, Germany, the United Kingdom, the Netherlands, Portugal), North America (Mexico, Canada, the United States), and South America (Brazil, Peru). The analyses of population coverage were suggestive of the fact that the designed vaccine candidates (MPXV-2 and MPXV-5) have the potential to combat the MPXV infection globally.

### 3.10 Immune simulation

**Figure 9:**
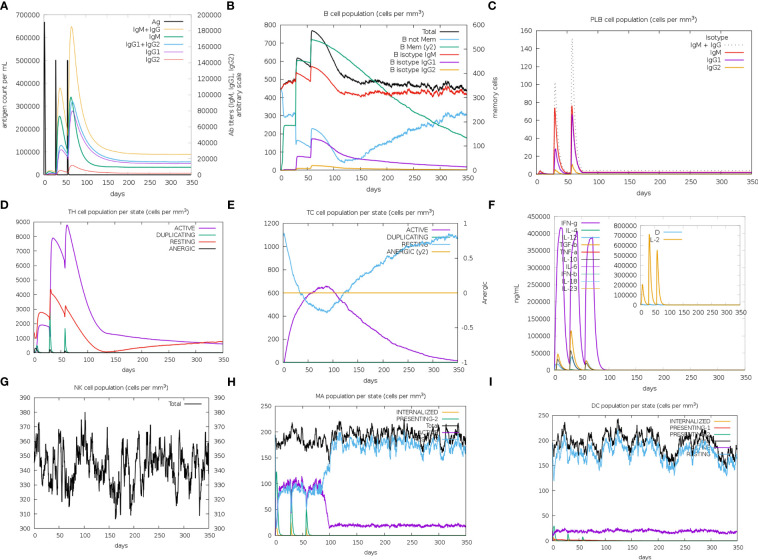
The plots relative to the immune stimulation of MPXV-5. **(A)** Titers of immunoglobulins and the immunocomplexes after vaccination. **(B)** Levels of B-cell population after vaccination. **(C)** Levels of plasma B-cell after vaccination. **(D)** Levels of helper T-cell cell population after vaccination. **(E)** Levels of cytotoxic T cells population after vaccination. **(F)** Concentration of cytokines and interleukins after vaccination. **(G)** Levels of NK cell population after vaccination. **(H)** Levels of MA population after vaccination. **(I)** Levels of DC population after vaccination.

The immune simulation results of MPXV-2, MPXV-5 and control group (C1) were shown in **Figure 9**; **Supplementary Figure 9**. Similar immune response patterns were observed between the simulations of candidate vaccines (MPXV-2 and MPXV-5) and C1. The primary response in the three simulations were both characterized by elevated immunoglobulin IgM (**Figure 9A**; **Supplementary Figure 9A**), B cells (isotype: lgM) (**Figure 9B**; **Supplementary Figure 9B**) and plasma cells (isotype: lgM) (**Supplementary Figure 9C**). In secondary and tertiary reactions, these elevations were elevated to a greater degree, and the elevation of different immunoglobulins and the immunocomplexes (IgM+IgG, IgG1+IgG2, IgG1, memory B cells and plasma cells (isotype: lgG1, lgG2) were also observed (**Figures 9B, C**; **Supplementary Figure 9B, C**). As shown in **Figures 9A–C**; **Supplementary Figure 9A–C** the titers of antibody (IgM + IgG, IgG1 + IgG2) and the level of plasma B cell (isotype: IgG1) in MPXV-2 group were higher than those in C1, while the titer of antibody (IgM + IgG, IgG1 + IgG2) and the level of plasma B cell (isotype: IgG1) in MPXV-5 group were lower than those in C1. Additionally, the helper T-cells along with corresponding memory cells were observed for two vaccine candidates and C1 (**Figure 9D**; **Supplementary Figure 9D**). The levels of active cytotoxic T cells and resting cytotoxic T cells in MPXV-2 and MPXV-5 groups changed significantly with time, while the resting cytotoxic T cell level in C1 changed little, and no inactive cytotoxic t cells were observed in the C1 group (**Figures 9E**; **Supplementary Figure 9E**). High levels of IFN-γ and IL-2 were observed in vaccine candidate groups and C1 group in the cytokines’ simulation plot (**Figure 9F**; **Supplementary Figure 9F**), while the concentration level of IL-2 was slightly higher for MPXV-2. During the immune simulation, the increased level of NK cells, macrophage (MA) and DC cells were also detected in three groups (**Figure 9G–I**; **Supplementary Figure 9G–I**). Together, the simulation results suggest that the MPXV-2 and MPXV-5 both can induce stronger immunity in the host body, thereby effectively combating the MPXV. Moreover, we can know that the humoral immune response provoked by MPXV-2 was much stronger than that of MPXV-5.

### 3.11 Codon optimization and *in silico* cloning

For the optimized codon sequences of MPXV-2 and MPXV-5 (Supplementary Data), the CAI were 0.97 and 0.94 respectively, and the GC content was 48.79% and 48.18%, respectively, showing that both the MPXV-2 and MPXV-5 might well express in *E. coli* K12 strain and have the potential for mass production. The optimized codon sequences of MPXV-2 and MPXV-5 were inserted into the pET28a (+) vector (**Supplementary Figure 10**). Electrophoretogram of enzyme digestion of pET28a (+)-MPXV-5 was shown in (**Supplementary Figure 11**).

## 4 Discussion

Since May 2022, monkeypox has broken the traditional prevalent situation, beginning to spread across several countries at the same time (4). However, no available therapeutics and preventions are available for MPXV infections highlighting the need for the development of a novel vaccine against MPXV. The multi-epitope vaccine can elicit specific and strong immune responses based on multiple conserved epitopes in several antigenic proteins, avoiding adverse reactions against undesirable epitopes (88). The application of immunoinformatics methods to design multi-epitope vaccines is becoming increasingly popular, which can significantly save time and expense in vaccine development. The selection of the target protein will deeply affect the vaccine’s efficacy. A35R, B6R and H3L, which were needed for viral attachment and entry and targeted by neutralizing antibodies (nAbs), were potential targets for diagnostic tests and vaccine/drug development (89). It is well known that mutations in the strain will weaken the effectiveness of the vaccine. Hence, to design a vaccine as effective as possible against the current epidemic MPXV strains and most MPXV strains, we contrived a multi-epitope vaccine from three reference strains of monkeypox at different epidemic stages as objects and A35R, B6R and H3L as targets.

Polypeptide-combined T-cell and B-cell epitopes often equip much stronger immunogenicity than T-cell or B-cell epitopes alone. So, to maximize the immunogenicity of the designed vaccine, every vaccine construct involved both T-cell epitopes (CTL and HTL epitopes) and B-cell epitopes of each target protein. Moreover, all selected epitopes were antigenic, non-allergenic, non-toxic and non-homologous with the human proteome, indicating that the multi-epitope vaccine can induce safe antigenic response but no cross-reaction with proteins in humans. Cytokines are a category of signaling molecules that play an important role in mediating and regulating immunity and inflammation. IFN-γ, IL-4 and IL-2 are several important cytokines produced by the immune system, which contribute to the differentiation of T cells and mediate cytotoxicity and antibody production (26–28). Hence, all antigenic HTL epitopes were assessed for the potential to induce the IFN-γ, IL-2 and IL-4 secretion, and only HTL epitopes that were capable of mediating the release of IFN-γ and IL-4 or IL-2 were considered for vaccine construction. Notably, the molecular docking between the T-cell epitopes and MHC molecules was performed, and the docking results revealed that the T-cell epitopes could successfully bind to MHC molecules, thereby being recognized by the antigen-presenting cell.

To acquire the optional vaccine candidates, five adjuvants were applied in this study. Although five selected adjuvants have been widely used in the design of multi-epitope vaccines in silico (48–51) and have been shown to stimulate the host’s immune response in animal experiments (43, 45, 52–54), any of these adjuvants have not been used for human vaccination. In the future, we can perform experiments to compare the immune enhancement effects of the adjuvants used in human vaccination, such as aluminum hydroxide and MF59 (90), and these adjuvants on multi-epitope vaccines. After the analysis of predicted biophysical and biochemical features of each vaccine construct (MPXV-1–5), two antigenic, safe, stable, thermostable and hydrophilic and soluble upon expression vaccine constructs (MPXV-2 and MPXV-5) were regarded as excellent vaccine candidates and subjected to further analysis.

Molecular docking analysis revealed strong binding affinities between MPXV-2 and MPXV-5 with TLRs (TLR2 and TLR4) and MHC molecules, which confirmed the capacity of the multi-epitope vaccine to be recognized by the human immune system, inducing stable and strong immune responses. MD simulation analysis indicated the MPXV-2-receptors and MPXV-5-receptors complexes’ stability through varying environmental conditions, including changing pressure, temperature, and motion. The initial trajectory evaluations, including the calculation of RMSD, RG, RMSF and hydrogen bonds, all correspond to the high stability of the vaccine-TLR and vaccine-MHC complexes in a biological environment. Gibb’s free energy analysis indicated that the MPXV-5-TLR4 had more stable conformations than other complexes. As shown in MM-PBSA calculations, the values of binding free energy of all complexes were negative, showing that all vaccine-receptor complexes could remain stable under natural conditions, especially the MPXV-5-TLR4 complex. Also, compared with other complexes, the binding mode of MPXV-5-TLR4 might be the most stable under natural conditions.

Notably, comparative immune stimulation was conducted between vaccine candidates and positive control. Similar immune response patterns were observed between the simulations of vaccine candidates (MPXV-2 and MPXV-5) and control group (C1), and the results of immune stimulation revealed that the MPXV-2 and MPXV-5 both can induce protective immune responses in the host. However, immune simulation performed by the C-IMMSIM server has simplified the process of antigen presentation. In general, the CTL and HTL epitopes are processed and presented by two distinct, proteasomal degradation (endogenous) and endo-lysosomal degradation (exogenous) pathways, respectively (91–93). The multi-epitope vaccine is considered an extracellular antigen. After being injected into the host body, the vaccine mainly activates CD4+T cells through the exogenous pathway to induce adaptive immunity and then activates B cells to generate humoral immunity. In the field of synthetic peptide vaccines, because the clinical benefits of early direct injection of synthetic peptides or the synthetic peptides loaded on DC cells are relatively inefficient, the use of complete proteins or long peptides to activate cellular immunity by cross-presentation had attracted more attention (94). The designed multi-epitope vaccines (MPXV-2 and MPXV-5) belong to long peptides. Therefore, the CTL epitopes from MPXV-2 and MPXV-5 can be cross-presented by DCs, thus activating CD8+T cells and generating cellular immunity. However, the cellular immunity induced by multi-epitope vaccines cross-presented by DC cells may not be strong enough. The high salt for mulation of Al(OH)3 can be selected as an adjuvant, which can significantly enhance the cross-presentation of extracellular antigens by DC cells (95). However, in the future, *in vitro* and *in vivo* experiments still need to verify that the multi-epitope vaccine with a suitable adjuvant can trigger an effective cellular immune response

## 5 Conclusion

Since May 2022, the United Kingdom, the United States, France, Spain, Brazil and other countries have successively reported their “first” cases of monkeypox, followed by a continuous increase in the number of cases. However, no specific treatment or vaccine for MPXV is currently available on the market. In this study, five multi-epitope vaccine constructs were developed against MPXV using immunoinformatics approaches, and the MPXV-2 and MPXV-5 were identified as the most promising vaccine constructs after the immunological and physiochemical analyses. The molecular docking and MD simulation confirmed that the MPXV-2 and MPXV-5 can form stable bindings to immune receptors and thereby trigger effective immune responses against MPXV. Additionally, the results obtained from immune simulation suggested that the MPXV-2 and MPXV-5 equip the capacity to elicit immune responses in the human body, but the antibody response induced by the MPXV-2 was stronger. Having said all this, the MPXV-2 and MPXV-5 could, in theory, effectively combat the MPXV.

## 6 Limitation

First, the immunogenicity of multi-epitope vaccines is weaker than that of inactivated or attenuated vaccines. The application of other available adjuvants, such as aluminum hydroxide, may address this issue (95, 96). Second, the immunogenicity of multi-epitope vaccines can be affected by the expression system, necessitating careful selection of the expression system during vaccine preparation. Although animal experiments using immunoinformatics to construct multi-epitope vaccines have shown promising results, and several vaccines designed using this approach are currently in clinical studies, as an emerging technology, more in-depth studies are still needed to verify its effectiveness. Finally, its protective effect needed to be confirmed by *in vitro* and *in vivo* experiments.

## Data availability statement

The original contributions presented in the study are included in the article/**Supplementary Material**, further inquiries can be directed to the corresponding author/s.

## Author contributions

CT, FZ, PP, AW and CL designed the research and analyzed the data; CT and FZ charged the drawing; CT, FZ, PP, AW and CL wrote the paper. PP, AW and CL reviewed and edited the paper. CL, AW and PP acquired the funding. All authors contributed to the article and approved the submitted version.
