# Supplementary material for: Development of multi-epitope vaccines against the monkeypox virus based on envelope proteins using immunoinformatics approaches
Source: Front Immunol. 2023 Mar 13;14:1112816. doi: 10.3389/fimmu.2023.1112816 (PMC10040844; doi:10.3389/fimmu.2023.1112816)
Supplement: Supplementary file 1 [file DataSheet_1.docx]

Supplementary Material

Development of Multi-Epitope Vaccines Against the Monkeypox Virus Based on Envelope Proteins Using Immunoinformatics Approaches

**Caixia Tan^1,6#^, Fei Zhu^2,3,4,5,6#^, Pinhua Pan^2,3,4,5,6*^, Anhua Wu^1,6*^, Chunhui Li^1,6*^**

*** Correspondence:** Chunhui Li: lichunhui@csu.edu.cn; Anhua Wu: xywuanhua@csu.edu.cn; Pinhua Pan: pinhuapan668@csu.edu.cn.

# Supplementary Data

## The DNA sequence of the MPXV-2

GGTGGTGGTTCTGGTATCATCAACACCCTGCAGAAATACTACTGCCGTGTTCGTGGTGGTCGTTGCGCTGTTCTGTCTTGCCTGCCGAAAGAAGAACAGATCGGTAAATGCTCTACCCGTGGTCGTAAATGCTGCCGTCGTAAAAAAGAAGCTGCTGCTAAAGCTAAATTCGTTGCTGCTTGGACCCTGAAAGCTGCTGCTGAAGCTGCTGCTAAAACCATGTCTGCTTTCCTGATCGTTCGTGCTTACTACTCTACCGAAACCTCTTTCAACGACAAAGCTTACTACGAAGCTACCTACCACATCATCATCATGGCTTACTACACCTACCACATCATCATCATGGCTCTGGCTTACTACTTCACCTACACCGGTGGTTACGACGTTGCTTACTACATCATGTTCATGCTGATCTTCAACGTTGCTTACTACATGTTCATGCTGATCTTCAACGTTAAACACGAATACGGTGCTGAAGCTCTGGAACGTGCTGGTACCCACCGTAAAGTTGTTTCTTCTACCACCCAGTACGACCACAAAGGTCCGGGTCCGGGTGAACAGGAAATCGAATCTCTGGAAGCTACCTACCACATCATCATCGGTCCGGGTCCGGGTGTTGTTATCATCGAAAACGACAACGTTATCGAAGACATCACCTTCGGTCCGGGTCCGGGTTCTGCTTACATCATCCGTGTTACCACCGCTCTGAACATCGTTGACGGTCCGGGTCCAGGAGGCTTCTACTTCGAAATAGCTCGTATCGAAAACGAAATGAAAATCGGTCCGGGTCCGGGTCCGAACTTCTGGTCTCGTATCGGTACCGTTGCTGCTAAACGTTACCACGAATACGGTGCTGAAGCTCTGGAACGTGCTGGTTGCATCCGTATCTCTATGGTTATCTCTCTGCTGTCTATGATCACCATGAAAAAACTGTCTATGATCACCATGTCTGCTTTCCTGATCGTTCGTCAGAACCAGAAAAAAAAACTGACCTCTACCGAAACCTCTTTCAACGACAAACAGAAAGTTACCAAAAAAAACGACGACCCGGACCACTACAAAGACTACGTTTTCATCCAGTGGACCAAAAAACACGACAAAAAAATCGACATCCTGCAGATGCGTGAAATCATCACCGGTAAAAAACCGAACTTCTGGTCTCGTATCGGTACCGTTGCTGCTAAACGTTACCCGAAAAAAATCCTGTTCATCATGTTCATGCTGATCTTCAACGTTAAATCTAAACTGCGTGTTCGTCGTCACCACCACCACCACCAC

## The DNA sequence of the MPXV-5

GGTGGTGGTTCTGCTCCGCCGCACGCTCTGTCTGAAGCTGCTGCTAAAGCTAAATTCGTTGCTGCTTGGACCCTGAAAGCTGCTGCTGAAGCTGCTGCTAAAACCATGTCTGCTTTCCTGATCGTTCGTGCTTACTACTCTACCGAAACCTCTTTCAACGACAAAGCTTACTACGAAGCTACCTACCACATCATCATCATGGCTTACTACACCTACCACATCATCATCATGGCTCTGGCTTACTACTTCACCTACACCGGTGGTTACGACGTTGCTTACTACATCATGTTCATGCTGATCTTCAACGTTGCTTACTACATGTTCATGCTGATCTTCAACGTTAAACACGAATACGGTGCTGAAGCTCTGGAACGTGCTGGTACCCACCGTAAAGTTGTTTCTTCTACCACCCAGTACGACCACAAAGGTCCGGGTCCGGGTGAACAGGAAATCGAATCTCTGGAAGCTACCTACCACATCATCATCGGTCCGGGTCCGGGTGTTGTTATCATCGAAAACGACAACGTTATCGAAGACATCACCTTCGGTCCGGGTCCGGGTTCTGCTTACATCATCCGTGTTACCACCGCTCTGAACATCGTTGACGGTCCGGGTCCAGGAGGCTTCTACTTCGAAATAGCTCGTATCGAAAACGAAATGAAAATCGGTCCGGGTCCGGGTCCGAACTTCTGGTCTCGTATCGGTACCGTTGCTGCTAAACGTTACCACGAATACGGTGCTGAAGCTCTGGAACGTGCTGGTTGCATCCGTATCTCTATGGTTATCTCTCTGCTGTCTATGATCACCATGAAAAAACTGTCTATGATCACCATGTCTGCTTTCCTGATCGTTCGTCAGAACCAGAAAAAAAAACTGACCTCTACCGAAACCTCTTTCAACGACAAACAGAAAGTTACCAAAAAAAACGACGACCCGGACCACTACAAAGACTACGTTTTCATCCAGTGGACCAAAAAACACGACAAAAAAATCGACATCCTGCAGATGCGTGAAATCATCACCGGTAAAAAACCGAACTTCTGGTCTCGTATCGGTACCGTTGCTGCTAAACGTTACCCGAAAAAAATCCTGTTCATCATGTTCATGCTGATCTTCAACGTTAAATCTAAACTGCGTGTTCGTCGTCACCACCACCACCACCAC

# Supplementary Figures

## Supplementary Figures 1


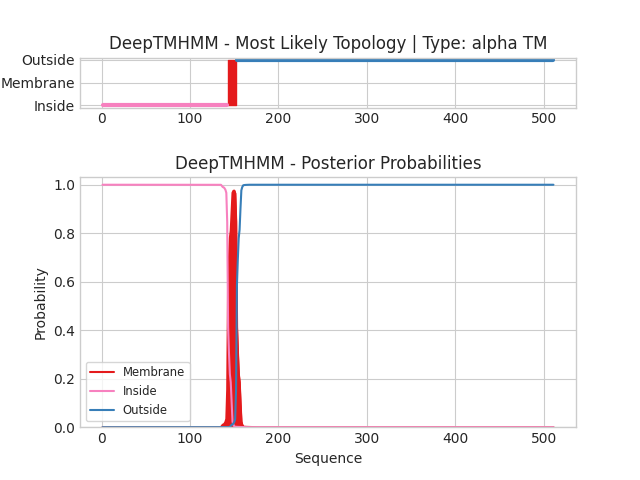


(a)


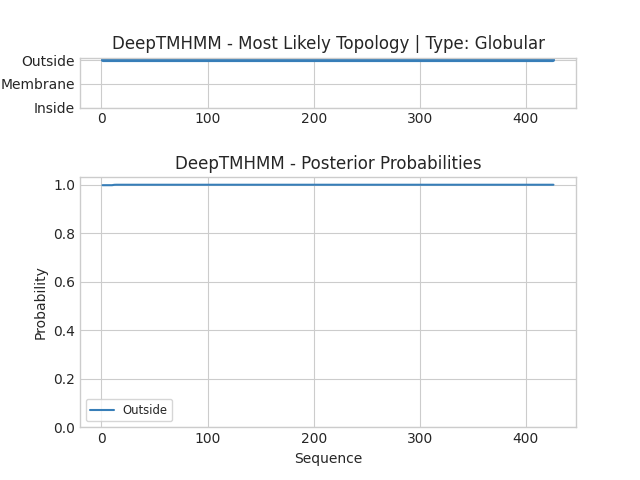


(b)


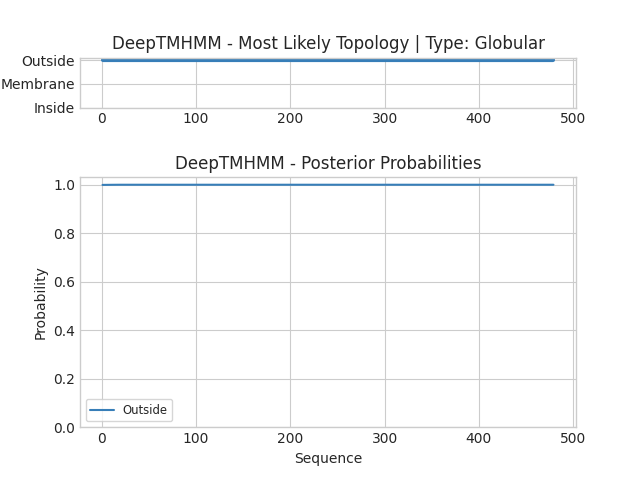


(c)


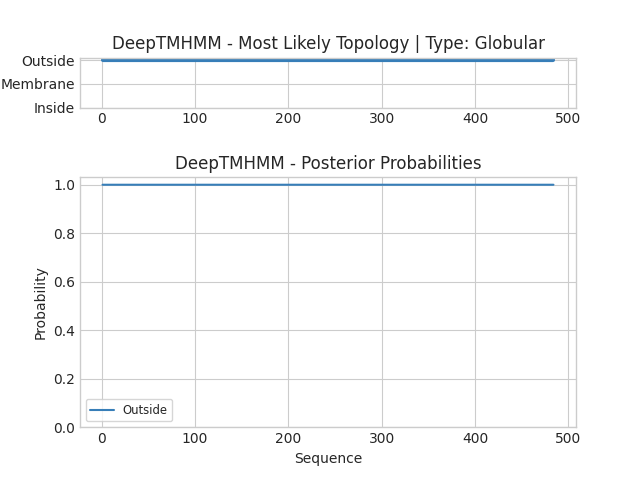


(d)


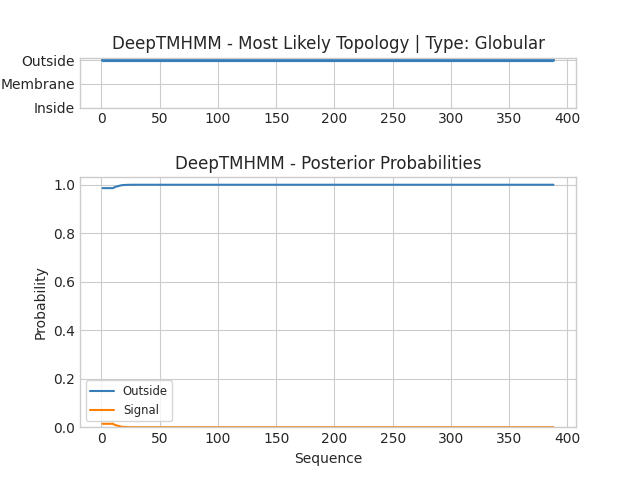


(e)

**Supplementary Figure 1.** The transmembrane helix of vaccine constructs;(a) MPXV-1; (b) MPXV-2; (c) MPXV-3; (d) MPXV-4; (e) MPXV-5.

## Supplementary Figures 2


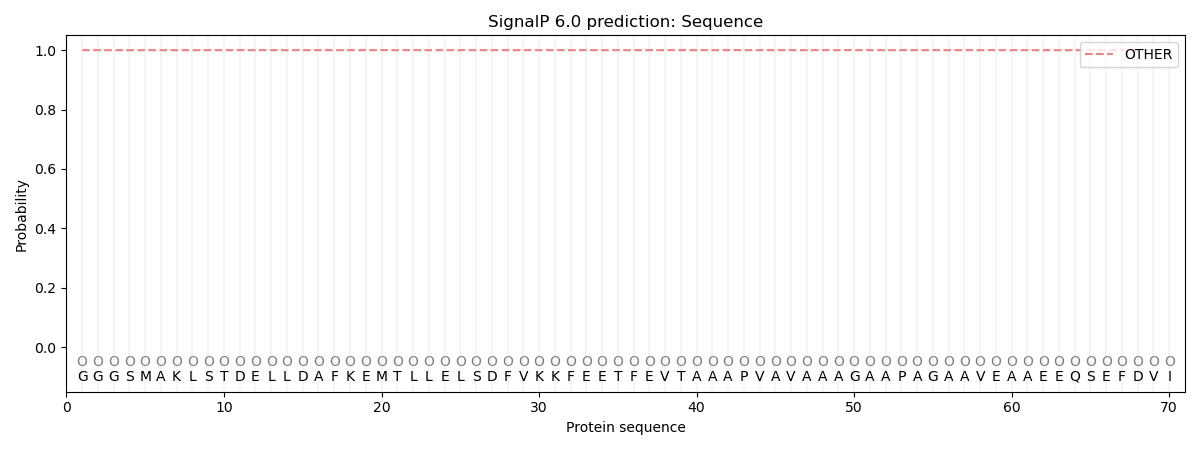


(a)


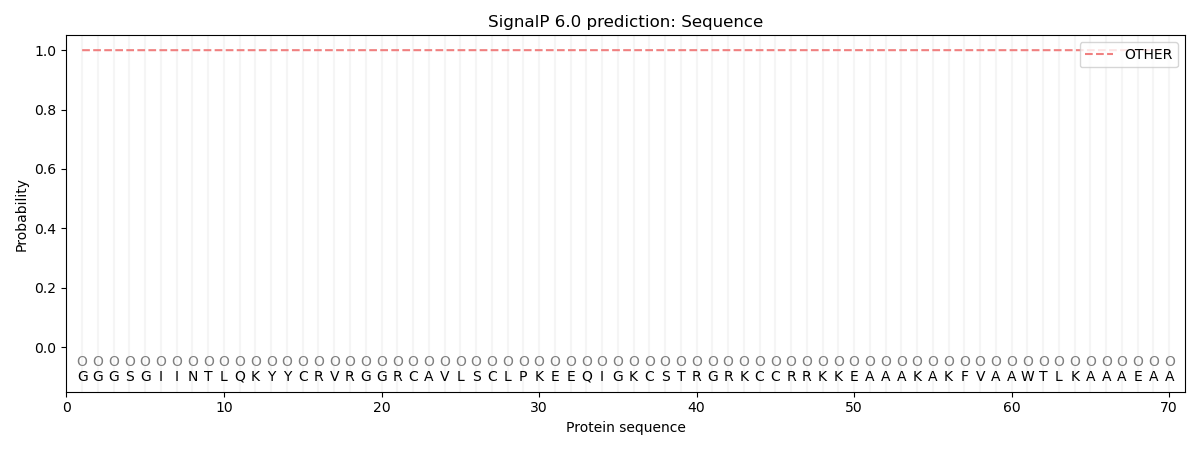


(b)


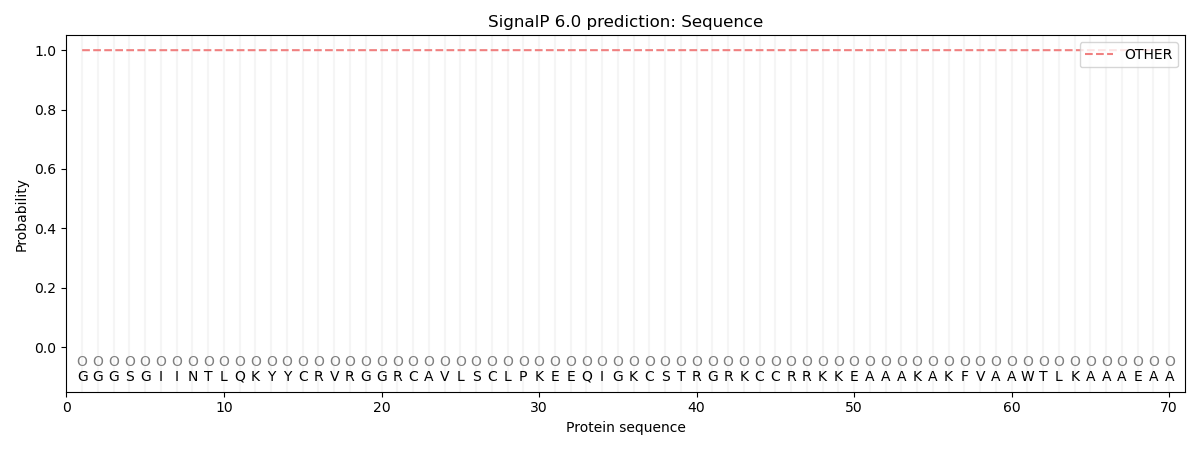


(c)


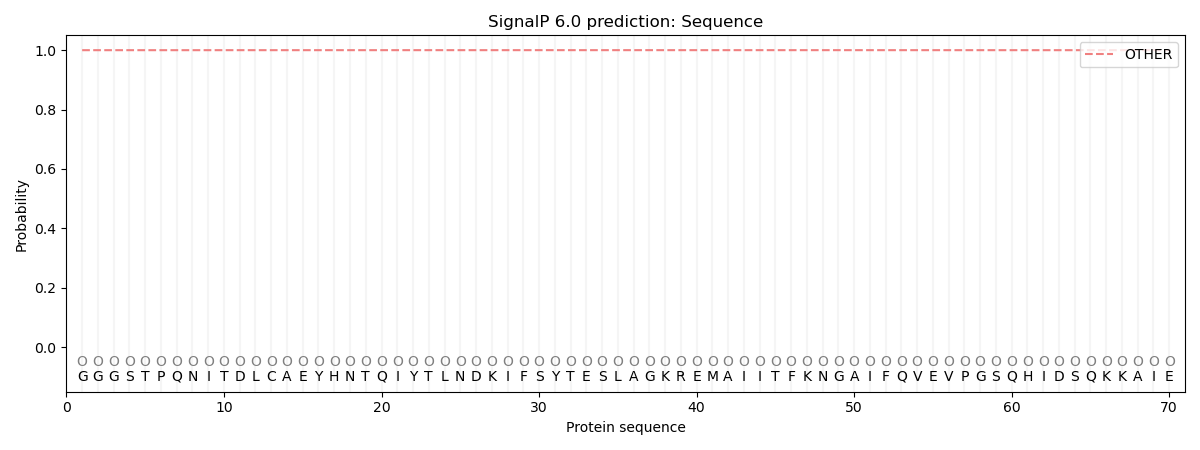


(d)


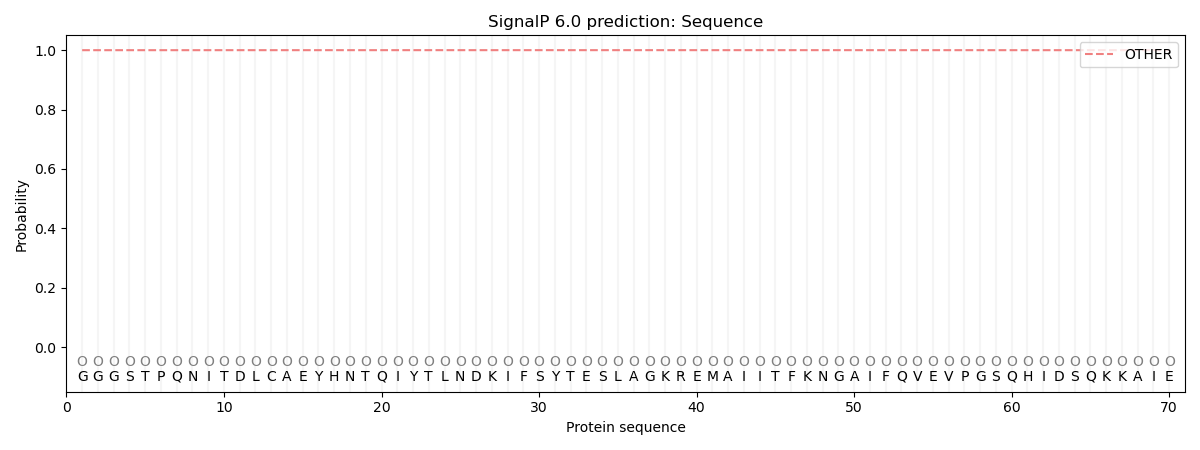


(e)

**Supplementary Figure 2.** The predicted signal peptide of vaccine constructs;(a) MPXV-1; (b) MPXV-2; (c) MPXV-3; (d) MPXV-4; (e) MPXV-5.

## Supplementary Figures 3


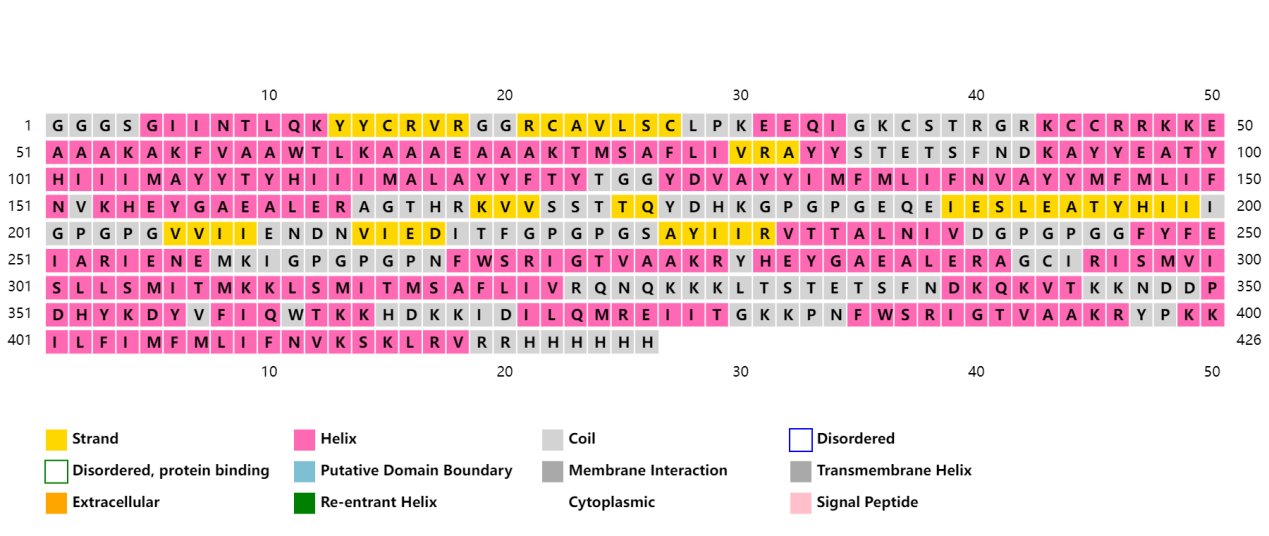
(a)


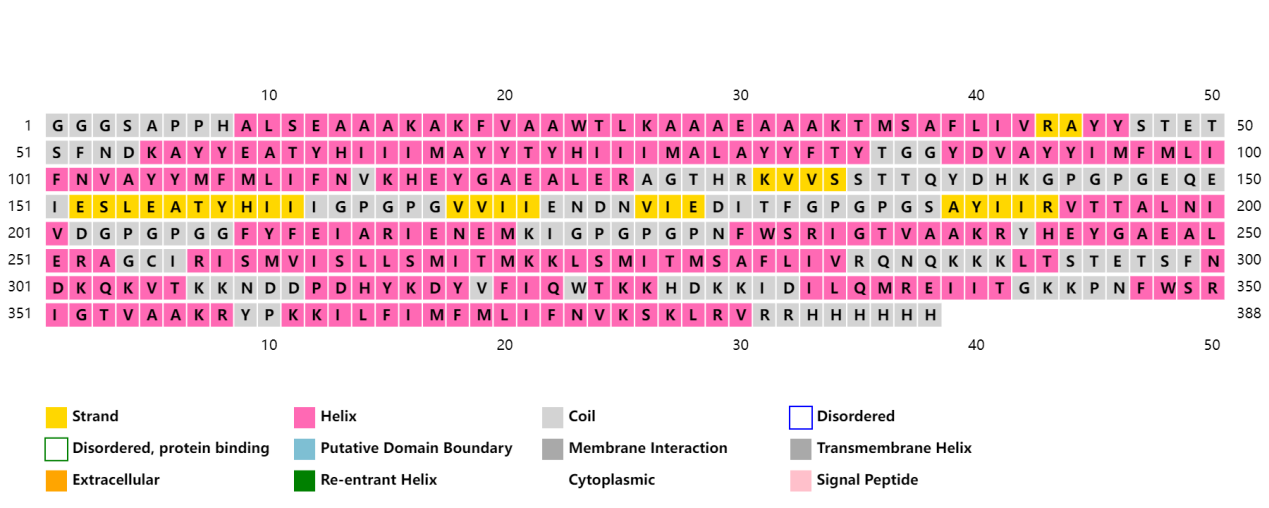
(b)

**Supplementary Figure 3**. The secondary structure of the vaccine sequence; (a) MPXV-2; (b) MPXV-5.

## Supplementary Figures 4


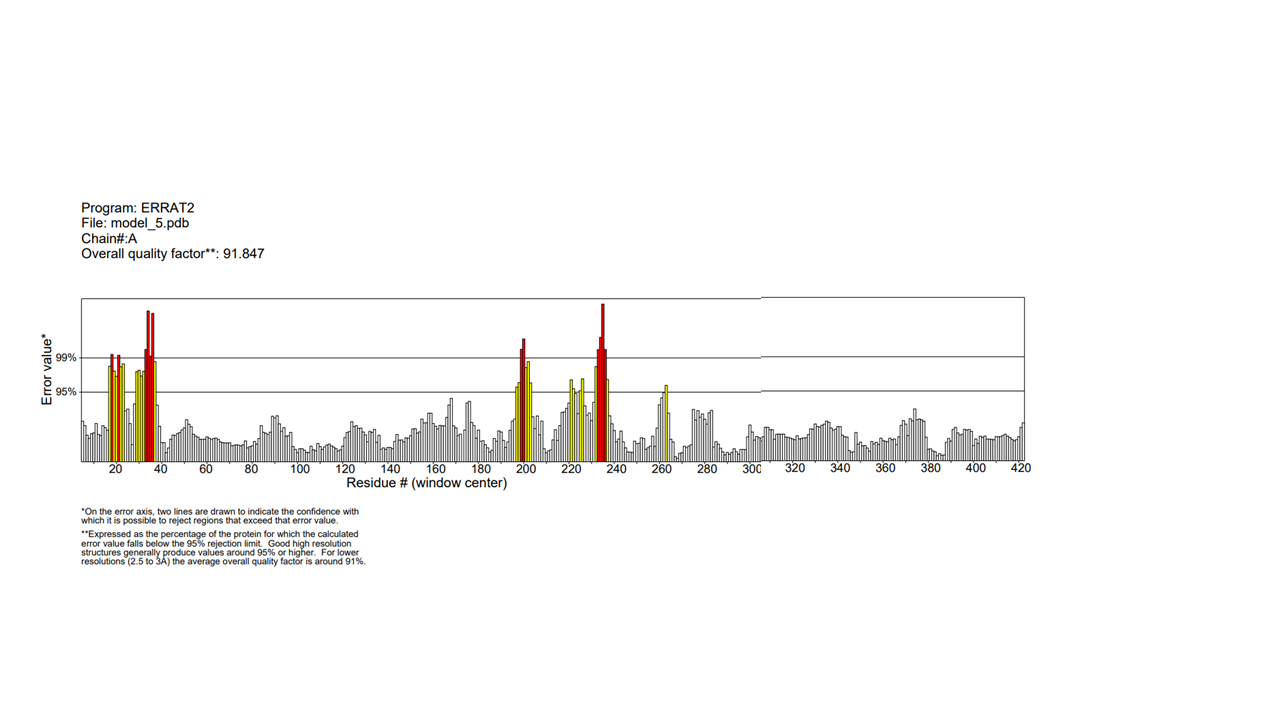
(a)


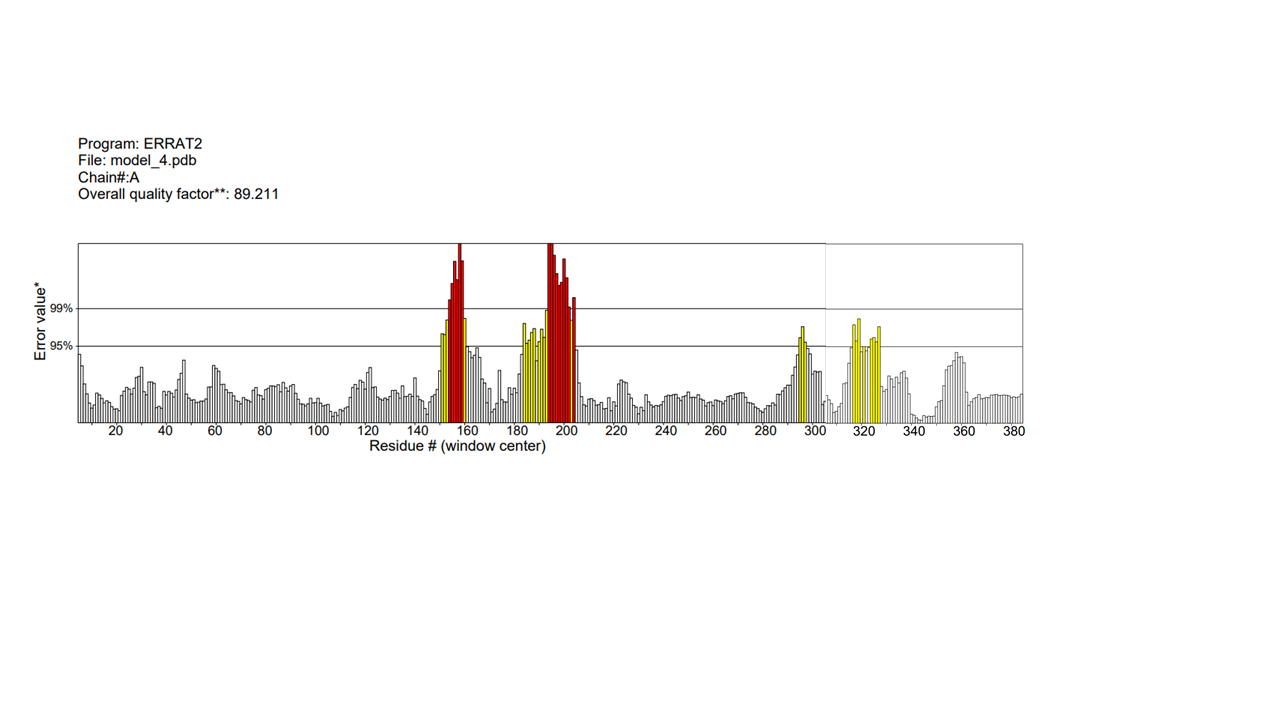


(b)

**Supplementary Figure 4**. The Overall Quality Factors generated by the ERRAT program of the vaccine’s refined tertiary structure; (a) MPXV-2; (b) MPXV-5.

## Supplementary Figures 5


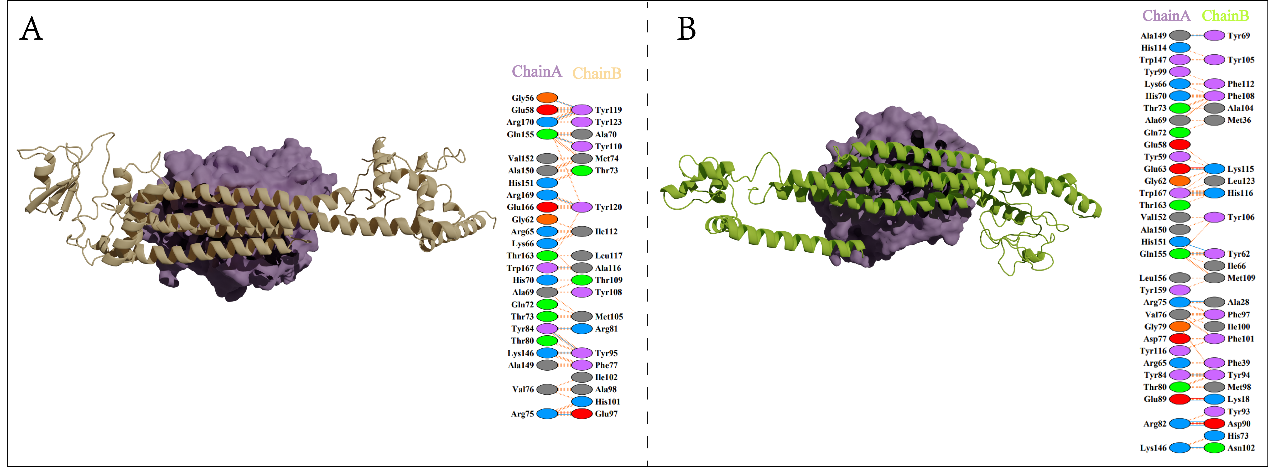


(a)


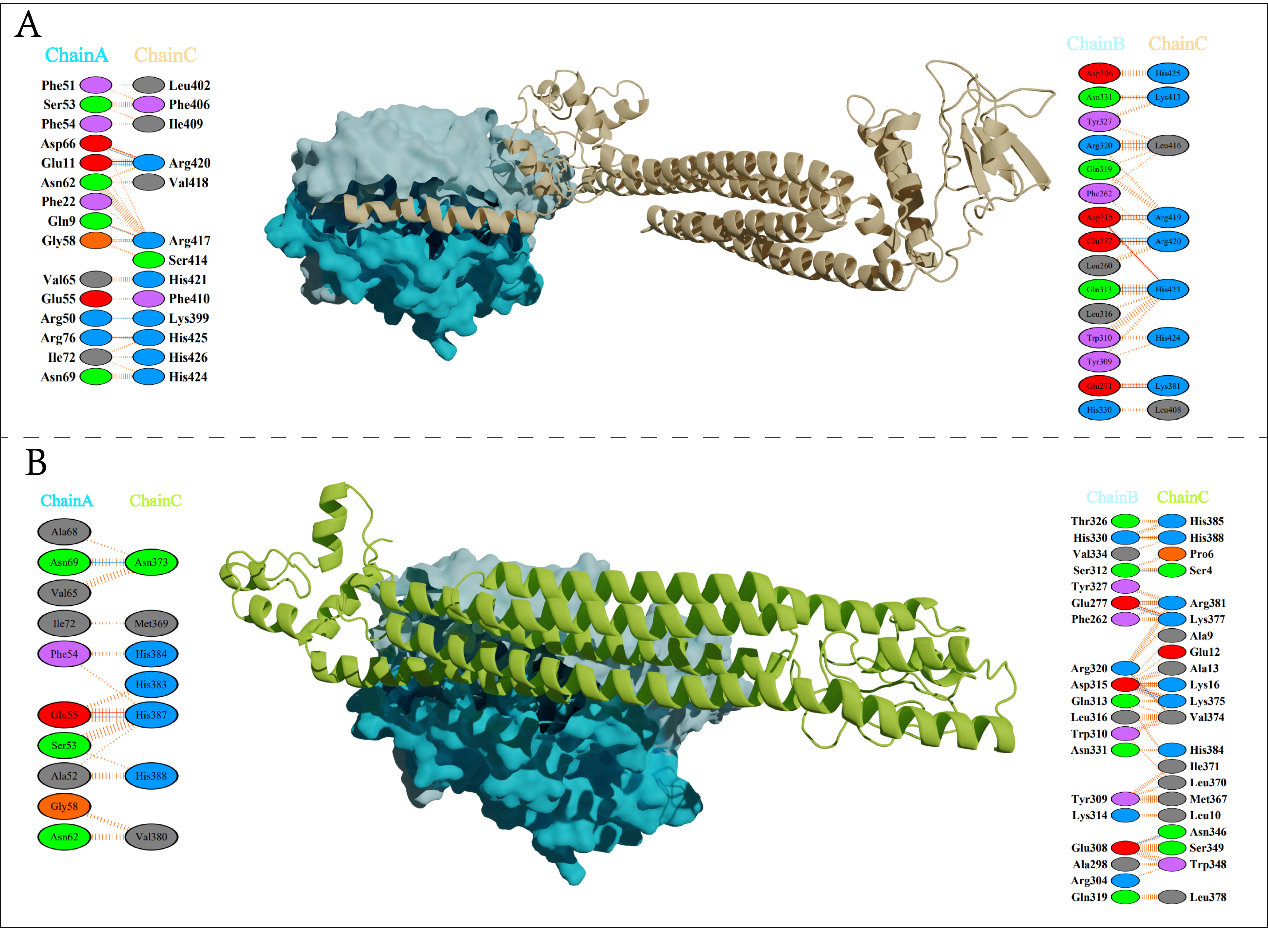


(b)

**Supplementary Figure 5**. Vaccine candidates (MPXV-2 and MPXV-5) docked with HLA-A*02：01 and HLA-DRB1*01:01. (a) Vaccine candidates (MPXV-2 and MPXV-5) docked with HLA-A*02：01; the plot on the left represents the binding mode between the MPXV-2 and HLA-A*02：01 and the interacting residues of the MPXV-2 with HLA-A*02:01; the plot on the right represents the binding mode between the MPXV-5 and HLA-A*02:01 and the interacting residues of the MPXV-5 with HLA-A*02:01; (b) Vaccine candidates (MPXV-2 and MPXV-5) docked with HLA-DRB1*01:01; the plot on the top represents the binding mode between the MPXV-2 and HLA-DRB1*01:01 and the interacting residues of the MPXV-2 with HLA-DRB1*01:01; the plot on the bottom represents the binding mode between the MPXV-5 and HLA-DRB1*01:01 and the interacting residues of the MPXV-5 with HLA-DRB1*01:01.

## Supplementary Figures 6


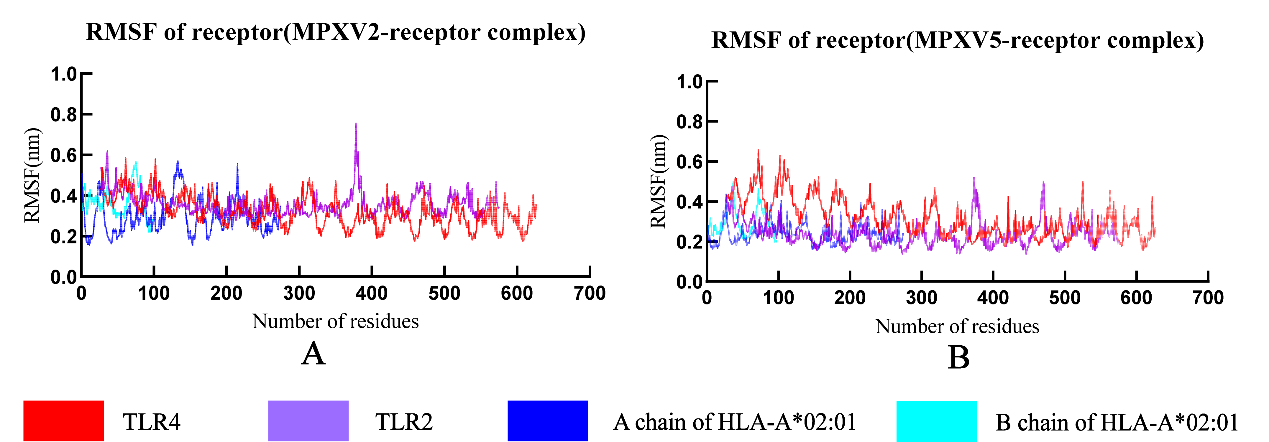


**Supplementary Figure 6**. The plot of Root means square fluctuation (RMSF) of receptor in vaccine-receptors complexes during the Molecular dynamics (MD) simulation. (A) RMSF of TLR2, TLR4 and HLA-A*02:01 chain in MPXV-2-TLR2, MPXV-2-TLR4 and MPXV-2-HLA-A*02:01 complexes; (B) RMSF of TLR2, TLR4 and HLA-A*02:01 chain in MPXV-5-TLR2, MPXV-5-TLR4 and MPXV-5-HLA-A*02:01 complexes;

## Supplementary Figures 7


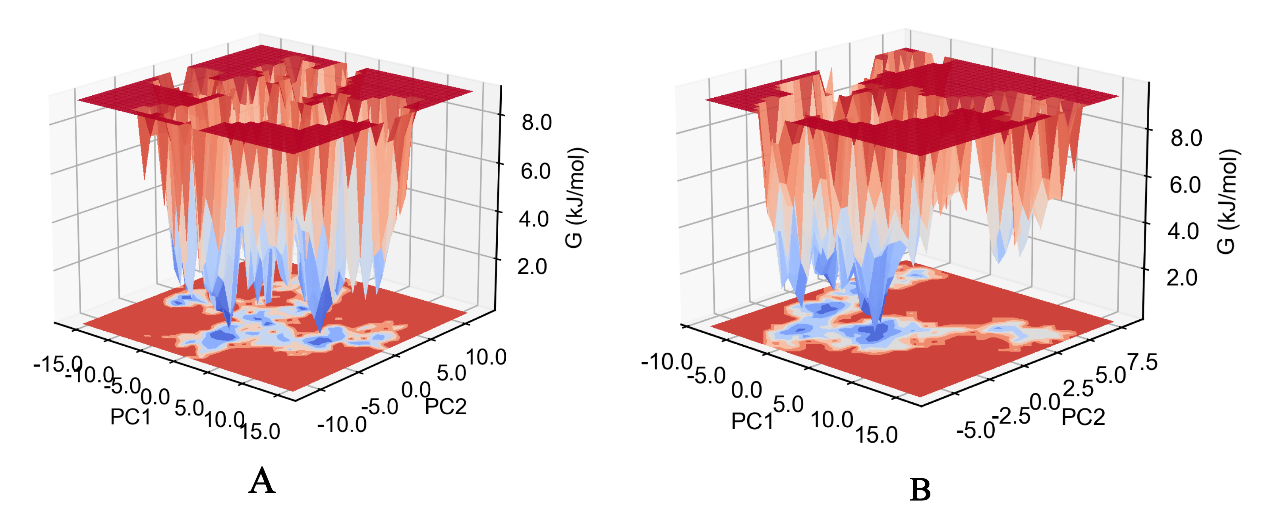


(a)


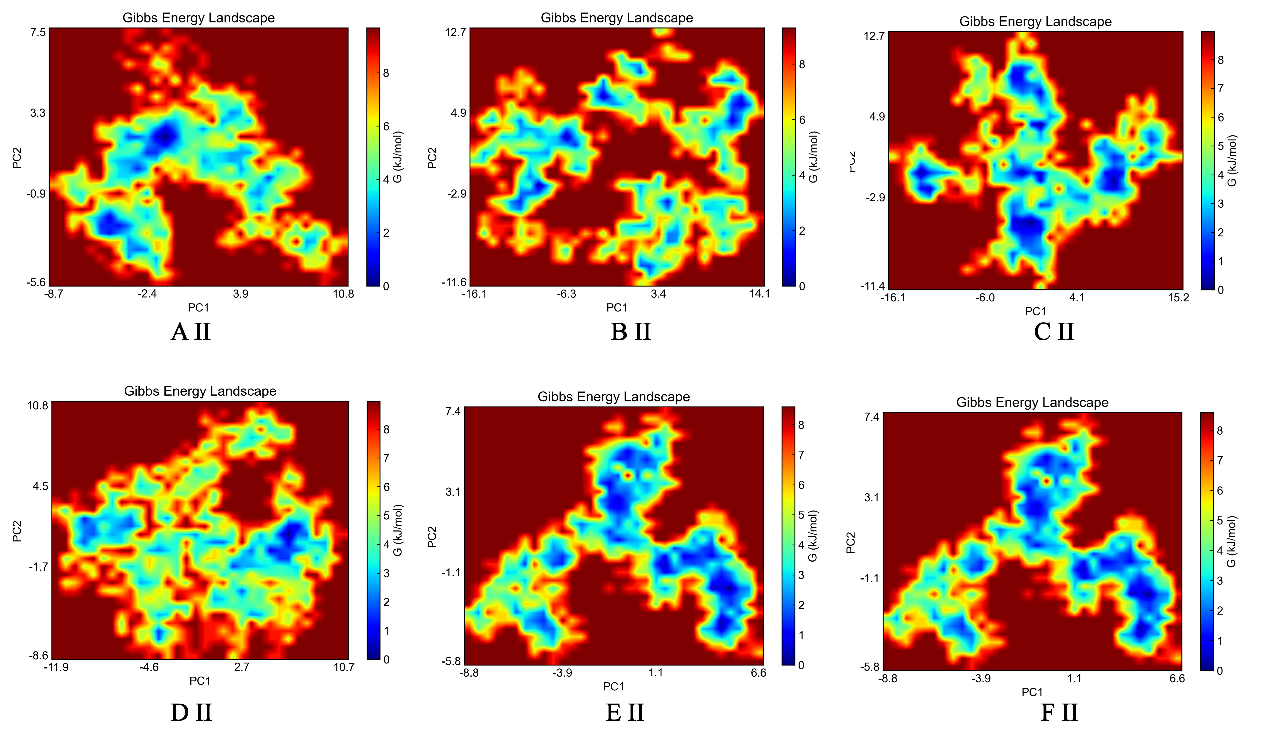


(b)

**Supplementary Figure 7**. Gibb’s free energy landscape. (a) three-dimensional of Gibb’s free energy landscape: (A) MPXV-2-HLA-A*02:01 complex. (B)MPXV-5-HLA-A*02:01 complex; (b) two dimensional of Gibb’s free energy landscape: (A Ⅱ) MPXV-2-TLR2 complex. (B Ⅱ) MPXV-2-TLR4 complex. (C Ⅱ) MPXV-2-HLA-A*02:01 complex. (D Ⅱ) MPXV-5-TLR2 complex; (E Ⅱ) MPXV-5-TLR4 complex; (F Ⅱ) MPXV-5-HLA-A*02:01 complex;

## Supplementary Figures 8


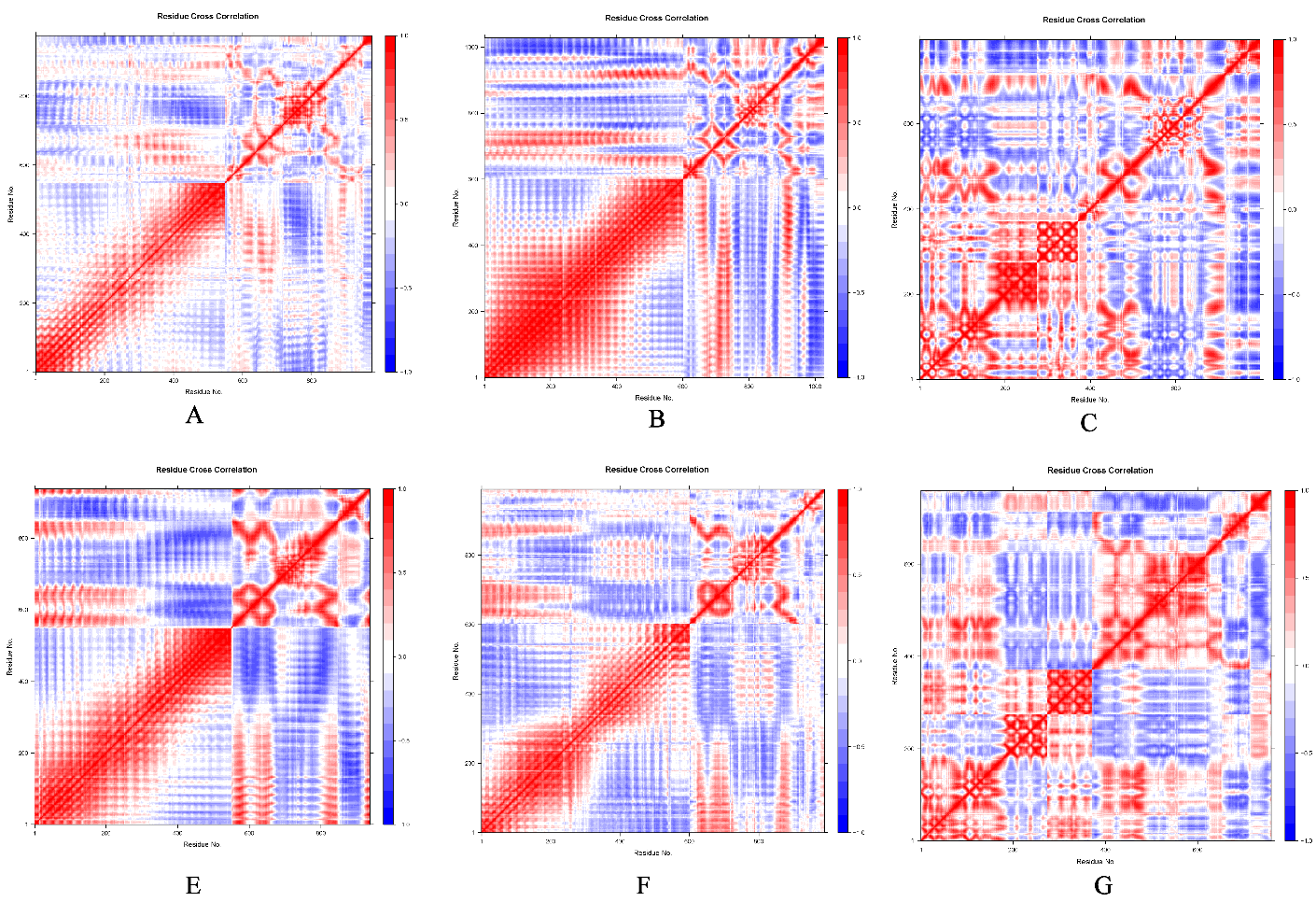


**Supplementary Figure 8.** Dynamic cross-correlation matrix (DCCM) analysis. (A) MPXV-2-TLR2 complex. (B) MPXV-2-TLR4 complex. (C) MPXV-2-HLA-A*02:01 complex. (D) MPXV-5-TLR2 complex. (E) MPXV-5-TLR4 complex. (C) MPXV-5-HLA-A*02:01 complex.

## Supplementary Figures 9


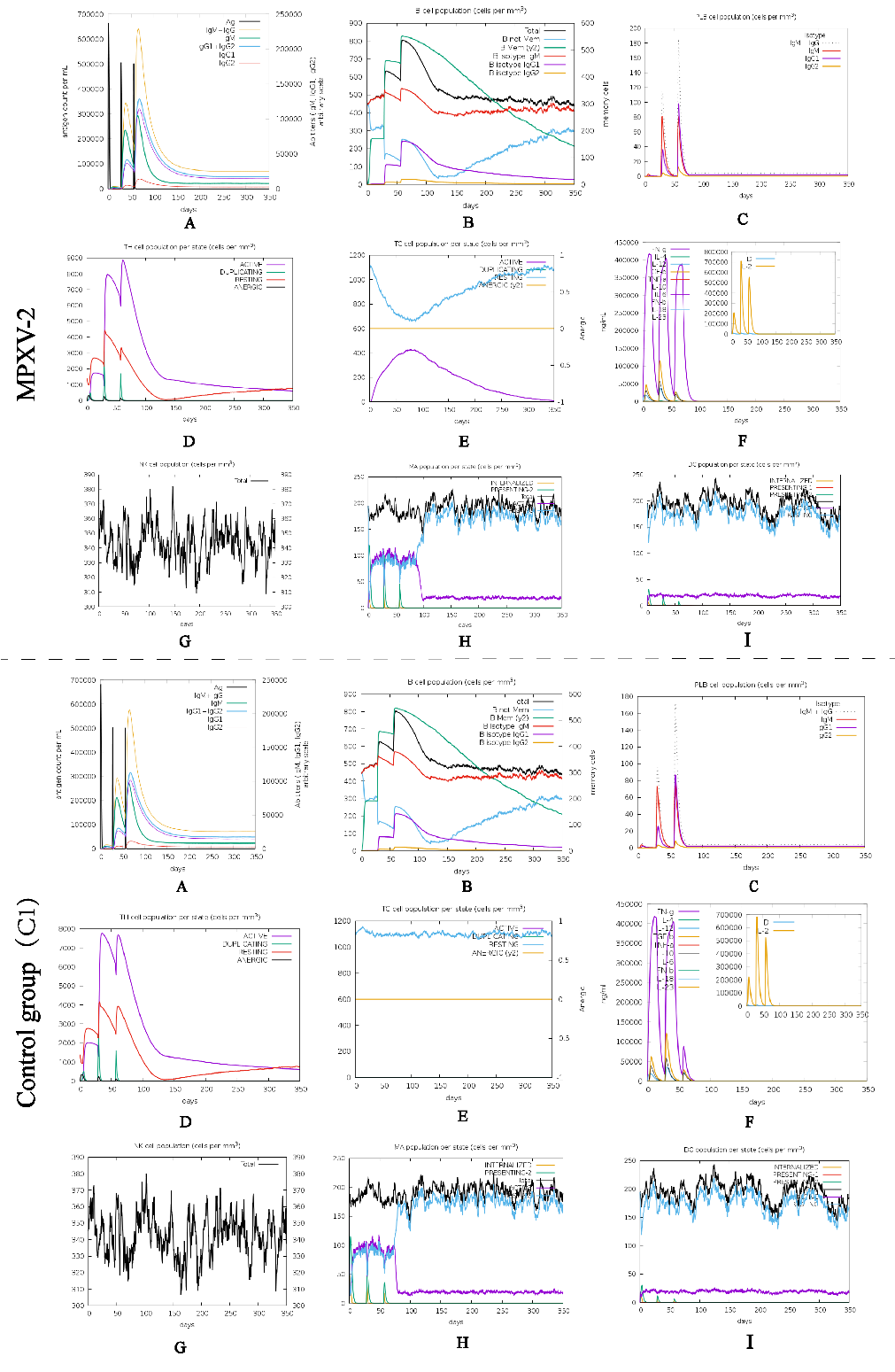


**Supplementary Figure 9**. The plots relative to the immune stimulation of MPXV-2 and the control group (C1). (A) Titers of immunoglobulins and the immunocomplexes after vaccination. (B) Levels of B-cell population after vaccination. (C)Levels of plasma B-cell after vaccination. (D) Levels of helper T-cell cell population after vaccination. (E) Levels of cytotoxic T cell population after vaccination. (F) Concentration of cytokines and interleukins after vaccination. (G) Levels of NK cell population after vaccination. (H) Levels of MA population after vaccination. (I) Levels of DC population after vaccination.

## Supplementary Figures 10


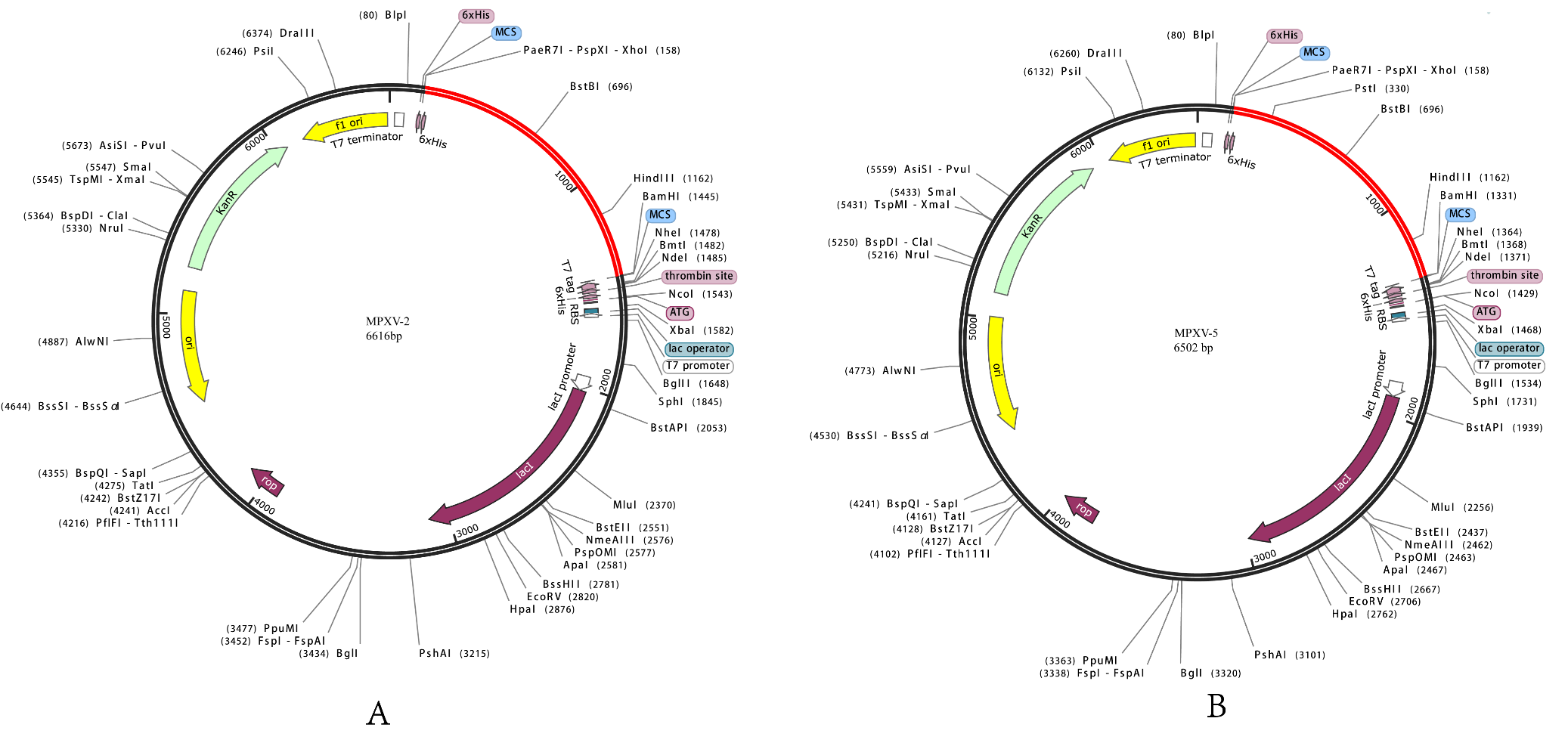


**Supplementary Figure 10**. The optimized codon sequences of the vaccines. (a) MPXV-2;(b) MPXV-5.

## Supplementary Figures 11


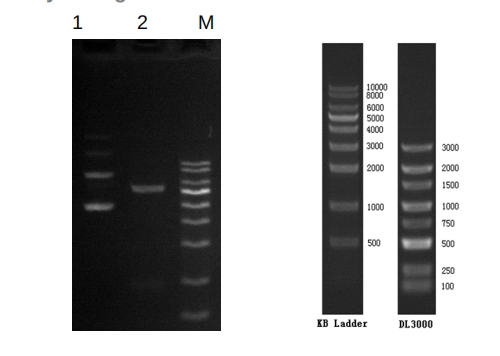


**Supplementary Figure 11**. The electrophoretogram of enzyme digestion of pET28a (+)-MPXV-5;1: Electrophoresis of the original plasmid; 2: Enzyme (HindIII and XhoI), Expected Size:1004/5498; M: Marker.
